# Natural Transformation Facilitates Transfer of Transposons, Integrons and Gene Cassettes between Bacterial Species

**DOI:** 10.1371/journal.ppat.1002837

**Published:** 2012-08-02

**Authors:** Sara Domingues, Klaus Harms, W. Florian Fricke, Pål J. Johnsen, Gabriela J. da Silva, Kaare Magne Nielsen

**Affiliations:** 1 Centre of Pharmaceutical Studies, Faculty of Pharmacy, University of Coimbra, Coimbra, Portugal; 2 Department of Pharmacy, Faculty of Health Sciences, University of Tromsø, Tromsø, Norway; 3 Institute for Genome Sciences, University of Maryland, School of Medicine, Baltimore, Maryland, United States of America; 4 Genøk-Centre for Biosafety, Tromsø, Norway; Harvard Medical School, United States of America

## Abstract

We have investigated to what extent natural transformation acting on free DNA substrates can facilitate transfer of mobile elements including transposons, integrons and/or gene cassettes between bacterial species. Naturally transformable cells of *Acinetobacter baylyi* were exposed to DNA from integron-carrying strains of the genera *Acinetobacter*, *Citrobacter*, *Enterobacter*, *Escherichia*, *Pseudomonas*, and* Salmonella* to determine the nature and frequency of transfer. Exposure to the various DNA sources resulted in acquisition of antibiotic resistance traits as well as entire integrons and transposons, over a 24 h exposure period. DNA incorporation was not solely dependent on integrase functions or the genetic relatedness between species. DNA sequence analyses revealed that several mechanisms facilitated stable integration in the recipient genome depending on the nature of the donor DNA; homologous or heterologous recombination and various types of transposition (Tn*21*-like and IS*26*-like). Both donor strains and transformed isolates were extensively characterized by antimicrobial susceptibility testing, integron- and cassette-specific PCRs, DNA sequencing, pulsed field gel electrophoreses (PFGE), Southern blot hybridizations, and by re-transformation assays. Two transformant strains were also genome-sequenced. Our data demonstrate that natural transformation facilitates interspecies transfer of genetic elements, suggesting that the transient presence of DNA in the cytoplasm may be sufficient for genomic integration to occur. Our study provides a plausible explanation for why sequence-conserved transposons, IS elements and integrons can be found disseminated among bacterial species. Moreover, natural transformation of integron harboring populations of competent bacteria revealed that interspecies exchange of gene cassettes can be highly efficient, and independent on genetic relatedness between donor and recipient. In conclusion, natural transformation provides a much broader capacity for horizontal acquisitions of genetic elements and hence, resistance traits from divergent species than previously assumed.

## Author Summary

Genetic elements, such as transposons and integrons, frequently carry antimicrobial resistance determinants and can be found widely disseminated among pathogenic bacteria. Their distribution pattern suggests dissemination through horizontal gene transfer. The role of natural transformation in horizontal transfer of genetic elements other than those that are self-replicative (plasmids) has remained largely unexplored. We have tested if natural transformation can facilitate transfer of transposons and class 1 integrons between bacterial species. We here provide experimental evidence showing that natural transformation can be a general mechanism for dissemination of genetic elements that by themselves do not encode interspecies transfer functions (e.g. transposons, insertion sequences). We demonstrate that antibiotic resistance determinants present in such genetic elements can spread by natural transformation between species of clinical interest. We show by quantitative data that interspecies exchange of resistance gene cassettes is highly efficient among integron-containing strains and species. Our study also provides a plausible explanation for how sequence-conserved integrons can become distributed among bacterial species.

## Introduction

The acquisition and dissemination of antibiotic resistance in Gram-negative bacteria is frequently facilitated by integrons [1]–[3]. Integrons contain genetic determinants for site-specific recombination and promoters driving the expression of gene cassettes [4]; the integrase (IntI) encoded by the integron facilitates site-specific acquisitions and excisions of gene cassettes within the integron [5]–[8]. The gene cassettes often encode antibiotic resistance, however cassettes conferring other metabolic functions to bacteria have also been described [9]–[11]. Integrons and integrases have been found to be present in approximately 9 to 17% of the sequenced bacterial genomes [1], [12]. Class 1 integrons are the most widely disseminated type in commensals and pathogens of human and animal origins [13]–[18], and have also been found in soil and in aquatic ecosystems [19]–[23]. This class of integrons is characterized by two conserved regions, the 5′ conserved segment (5′-CS), which includes the integrase gene (*intI1*), the adjacent recombination site (*attI1*) and the promoter (Pc), and the 3′ conserved segment (3′-CS), which contains the *qacEΔ1* gene (encoding an incomplete version of a quaternary ammonium compound resistance), the *sulI* (encoding resistance to sulfonamides) and the *orf5* [2], [24].

Highly similar class 1 integrons have been found in both Gram-negative and -positive bacteria [25], [26] and integrons with the same composition and organization have been found in unrelated bacterial species and strains in geographically distinct areas [27]–[30]. The exceptional broad potential of integrons to disseminate among pathogenic bacterial strains and species is remarkable because they only harbor functions for genomic integration and excision and do not encode functions that enable horizontal transfer between bacterial cells. Class 1 integrons are often present in plasmids, transposons and insertion sequences [12], [31], [32], and their dissemination is considered to depend on horizontal mobility of the genetic element or the genetic region they reside drawing on both transduction and conjugational processes [33]–[35]. However, horizontal movement of class 1 integrons genetically linked to non-conjugative elements [36] or incomplete mobile genetic elements and transposons [20], [32], [37] remains to be explained. Only few studies have attempted to experimentally examine how (non-conjugative) transposons, integrons or gene cassettes move horizontally. These include reports on the recruitment of gene cassettes [38], the acquisition and integration of synthetic gene cassettes by natural transformation [39] and transposition of the integron In33 [40], [41]. Horizontal transfer of non-conjugative transposons seems to rely on linkage to conjugative elements or on transduction by bacteriophages [42], [43].

Here we have investigated the potential for transposons, integrons and gene cassettes, supplied as fragmented DNA substrates, to move horizontally between bacterial species via natural transformation. Our model system relied on exposing the transposon- and integron-free and naturally-transformable bacterium *Acinetobacter baylyi* [44]–[46] to purified DNA or cell lysates obtained from the integron-carrying Gram-negative bacteria: *A. baumannii*, *Citrobacter freundii*, *Enterobacter cloacae*, *Escherichia coli*, *Escherichia fergusonii*, *Pseudomonas aeruginosa*, *Salmonella enterica* serovar Rissen and serovar Typhimurium. The exposure of naturally competent *A. baylyi* cells to DNA from these sources led to the acquisition of novel resistance traits as well as entire integrons and transposons. Transposition-based integration occurred between unrelated hosts, whereas both transposition and homologous recombination facilitated acquisitions from related host species.

Both the donor strains and the transformant isolates were characterized by antibiotic resistance profiling, targeted PCR, DNA sequencing by extensive primer walking, genome sequencing of two transformants, pulse-field gel-electrophoresis (PFGE) and Southern blot hybridization. A pairwise growth competition assay was undertaken to determine the impact of the acquired integrons on relative fitness. The integron-carrying transformants of *A. baylyi* were also used in subsequent transformation assays to confirm the nature of the initial interspecies gene transfer and determine further intraspecies transfer frequencies.

## Materials and Methods

### Bacterial strains and cultures

The naturally competent soil bacterium *A. baylyi* BD413 (spontaneous rifampicin resistant mutant) [47] and close derivatives (this work) were used as recipients. A highly similar strain (ADP1) has been sequenced (acc. no. CR543861) [44]; only few differences between the two laboratory strains are expected [48]. The integron-carrying bacteria were *A. baumannii* 064, *A. baumannii* 65FFC, *P. aeruginosa* SM, all clinical isolates, *S. enterica* serovar Rissen 486 and *S. enterica* serovar Typhimurium 490, both isolated from pork processed food, and *C. freundii* C16R385, *E. cloacae* C2R371, *E. coli* C10R379, and *E. fergusonii* AS041A2 isolated from food-producing and wild animals (Table 1).

**Table 1:** Summary of the strains used in this study.

Strain	Origin	Plasmid	Integron size^a^ (bp)	Gene cassettes	Relevant phenotype/trait^b^	Reference
DNA donors:						
*Acinetobacter baumannii* 064	Clinical	Yes	762	*aadB*	K^R^	This study
*A. baumannii* 65FFC	Clinical	No	1000	*bla_IMP-5_*	CTX^R^	[28]
*A. baylyi* SD1	Transformant (DNA of *S. enterica* 490)	No	2000	*bla_OXA-30_+aadA1*	SC^R^	This study
*A. baylyi* SD2	Transformant (DNA of *A. baumannii* 064)	No	762	*aadB*	K^R^	This study
*A. baylyi* SD3	Transformant (DNA of *A. baumannii* 065FFC)	No	1000	*bla_IMP-5_*	CTX^R^	This study
*A. baylyi* SD4	Transformant (DNA of *S. enterica* 490	No	2000	*bla_OXA-30_+aadA1*	SC^R^	This study
*A. baylyi* SD5	Transformant (DNA of *S. enterica* 486)	No	1912	*dfrA12+aadA2*	SC^R^	This study
*A. baylyi* SD6	Transformant (DNA of *P. aeruginosa* SM)	No	3000	*aacA4+bla_PSE_+aadA2*	SC^R^	This study
*A. baylyi* [KOI](Ps)1	Transformant (DNA of *P. aeruginosa* SM)	No	3000	*aacA4+bla_PSE_+aadA2*	SC^R^; *intI1*::*cat*	This study
*Citrobacter freundii* C16R385	Rabbit	Yes	1913	*dfrA12+orfF+aadA2*	SC^R^	Domingues *et al.*, unpublished
*Enterobacter cloacae* C2R371	Rabbit	Yes	1913	*dfrA12+orfF+aadA2*	SC^R^	Domingues *et al.*, unpublished
*Escherichia coli* C10R379	Rabbit	No	769	*dfrA7*	W^R^	Domingues *et al.*, unpublished
*Escherichia coli* K71-77^c^	Clinical	Yes	n.d.	*-----*	CN^R^	[106]
*Escherichia fergusonii* AS041A2	Owl	Yes	1594	*dfrA1+aadA1*	W^R^	Domingues *et al.*, unpublished
*Klebsiella pneumoniae* K66-45^c^	Clinical	Yes	n.d.	*-----*	CN^R^	[106]
*Pseudomonas aeruginosa* K34-73^c^	Clinical	No	3604	*bla_VIM-4_+arr-7+aacA4+aadA1*	CN^R^	[107]
*P. aeruginosa* SM	Clinical	No	3000	*aacA4+bla_PSE_+aadA2*	SC^R^	[67]
*Salmonella enterica* serovar Rissen 486	Fresh pork sausage	Yes	1912	*dfrA12+aadA2*	SC^R^	This study
*S. enterica* serovar Thyp. 490	Pork hamburger	Yes	2000	*bla_OXA-30_+aadA1*	SC^R^	Da Silva, unpublished
Recipients:						
*Acinetobacter baylyi* BD413	Soil	No	No	-----	R^R^	[47], [108]
*A. baylyi* SD2	Transformant (DNA of *A. baumannii* 064)	No	762	*aadB*	K^R^	This study
*A. baylyi* KOI	Transformant (DNA of SD2)	No	762	*aadB*	K^R^; *intI1*::*cat*	This study
*A. baylyi* RAM	Transformant (DNA of SD2)	No	762	*aadB*	K^R^, *recA*::*cat*	This study
*A. baylyi* SD9	Transformant (DNA of BD413)	No	No	*-----*	Δ*recBCD* Δ*sbcCD*	This study
*A. baylyi* KOC4	Transformant (DNA of BD413)	No	No	*-----*	W^R^, Δ*comFECB*::*dhfr*	This study
Genome sequenced:						
*A. baylyi* (AbII)3	Transformant^d^	No	762	*aadB*	K^R^	This study
*A. baylyi* (St)3	Transformant^e^	No	2000	*bla_OXA-30_+aadA1*	SC^R^	This study

a Obtained with 5′-CS and 3′-CS primers [24].

b The resistance trait was used for selection in the natural transformation assays.

c Transformants obtained after exposure to these sources of DNA were only tested at the phenotypic level.

d Obtained by natural transformation of *A. baylyi* BD413 with purified DNA from *A. baumannii* 064. Integron acquisition was shown first by PCR, which explains the antimicrobial susceptibility profile of the tested antibiotics. The PFGE profile obtained with restriction digestion using the enzyme I-*Ceu*I revealed unexpected bands that co-hybridized with the probe for *intI*1 gene in Southern blot.

e Obtained by natural transformation of *A. baylyi* BD413 with purified DNA from *S. enterica* serovar Thypimurium 490. Integron acquisition was shown first by PCR, which explains the antimicrobial susceptibility profile of the tested antibiotics. Initial sequencing of the integron flanking regions, by primer walking, showed that the acquired fragment was large; suggestive of the possible acquisition of a plasmid (excluded after sequencing).

R resistance; CN – gentamicin, CTX – cefotaxime, K – kanamycin, R – rifampicin, SC – spectinomycin, W – trimethoprim.

n.d.- not determined.

The transformability of *A. baylyi* was also determined with DNA extracted from three clinical, multi-resistant strains *E. coli* K71-77, *Klebsiella pneumoniae* K66-45, both carrying the NDM-1 metallo-β-lactamase among other resistance genes, and *P. aeruginosa* K34-73, carrying the VIM-4 metallo-β-lactamase (Table 1). The NDM-1 genes of both *E. coli* K71-77 and *K. pneumoniae* K66-45 are plasmid-encoded; *P. aeruginosa* K34-73 carries a class 1 integron with four gene cassettes (*bla*_VIM-4_-*arr-7*-*aacA4*-*bla*_PSE-1_) in the chromosome. The transformants yielded from exposure to DNA from these 3 latter strains were only characterized at the phenotypic level.

Some of the transformants of *A. baylyi* that were confirmed to have taken up the integron from the heterologous donor sources were also used as a source of donor DNA in subsequent transformation experiments (i.e. isolates SD1, SD2, SD3, SD4, SD5 and SD6; see Table 1). One of the integron-carrying transformants of *A. baylyi* (isolate SD2) was also used as a recipient bacterium in subsequent transformation assays (see Table 1).

Other derivatives of the *A. baylyi* BD413 strain employed as recipients (Table 1) were SD9 (a Δ*recBCD* Δ*sbcCD* double mutant constructed as described by Harms and Wackernagel [49]) and the SD2 derivatives KOI (*intI1*::*cat*) and RAM (*recA*::*cat*) constructed as follows: an internal segment (724 bp) from the *intI1* gene of *A. baumannii* 064 was PCR-amplified using the primers intI1-f (5′-AGCTTACGAA CCGAACAGGC-3′) and INCINTF (5′-TGATGCCTGC TTGTTCTACG-3′) and Phusion DNA polymerase (Finnzymes, Finland), according to the manufacturer's instructions, and inserted into the *Sma*I site of pACYC177, resulting in pACYC177-int36. Next, a 1077 bp segment covering the *cat* (chloramphenicol resistance) gene from pACYC184 was amplified with primers cat-f (5′-CTCCGCTAGC GCTGATGTCC-3′) and cat-r (5′-GTAGCACCAG GCGTTTAAGG-3′) using Phusion polymerase and inserted into the singular *Pvu*II site located in the *intI1* segment of pACYC177-int36, resulting in pACYC177-int-cat. This plasmid was *Hin*cII-linearized and used to naturally transform *A. baylyi* SD2, giving strain KOI (verified by PCR). In parallel, an internal 938 bp segment of the *recA* gene of ADP1 was PCR-amplified with primers recA-f (5′-AGCAAGGCAT TACAAGCTGC-3′) and recA-r (5′-AATTCTGTAG AAATCTGAGG-3′) and Phusion and inserted into the *Hin*cII site of pUC19, giving pUC19-recA. The 1077 bp cat segment was cloned into the singular *Hin*cII site of pUC19-recA (located in the center of the *recA* fragment), resulting in pUC19-recA-cat which was *Xmn*I-linearized and used to inactivate *recA* of *A. baylyi* SD2 by natural transformation to yield strain RAM (verified by PCR). The non-transformable strain KOC4 was constructed by transformation of BD413 by DNA from a strain carrying a Δ*comFECB*::*dhfr* allele (trimethoprim-resistant) [50] (A. Utnes, unpublished data).

*A. baylyi* was cultivated in Luria-Bertani (LB) medium with rifampicin (R) 50 µg/ml; wild-type donor bacteria as well as transformants were grown, and selected for in LB supplemented with antibiotics according to their phenotype: ampicillin (AM; 25 µg/ml), cefotaxime (CTX; 10 µg/ml), chloramphenicol (Cl; 5 or 10 µg/ml), gentamicin (CN; 10 µg/ml), kanamycin (K; 10 µg/ml), R (25 µg/ml) spectinomycin (SC; 10 µg/ml) and trimethoprim (W; 250 µg/ml). *A. baylyi* cells and transformants were grown at 30°C, and the different donor bacteria at 37°C.

### DNA extraction

Genomic DNA used in the transformation assays (10 µg) was isolated from bacterial cultures using anion exchange columns (QIAGEN, Germany) columns according to the manufacturers protocol and resuspended in EB buffer, pH 8.5 (QIAGEN, Germany). Plasmid DNA was isolated using a Plasmid Mini Kit (QIAGEN, Germany). The DNA concentration was measured with a UV/VIS spectrophotometer (6405 Spectrophotometer, Jenway, England) or a Nanodrop ND-1000 (Nanodrop Technologies, USA).

For the preparation of supernatants (lysate) of the heat-treated bacterial cell suspensions, 5 ml overnight cultures of the bacteria were centrifuged at 20,000×g for 5 min and resuspended in water (50 µl), followed by heat treatment at 80°C for 15 min. The raw lysate was centrifuged, and the supernatant containing DNA was collected [51]. The lack of viable cells was confirmed by streaking aliquots on LB plates.

### Natural transformation assays

The recipient cells were prepared, and experiments were performed on nitrocellulose filters placed on agar-surfaces, as previously described [52], [53]. Each transformation assay was repeated between two and ten times (each assay was done in triplicate). Transformation assays were done with: i) *A. baylyi* BD413 as recipient and DNA extracted from various wild-type integron-carrying species; ii) with *A. baylyi* BD413 as recipient and DNA extracted from integron-carrying *A. baylyi* transformants; iii) with integron-carrying *A. baylyi* transformant SD2 as recipient and DNA extracted from various wild-type integron-carrying species; iv) with an integrase deletion carrying *A. baylyi* transformant (KOI) as recipient and DNA extracted from various wild-type integron-carrying species; v) with an integron-carrying *A. baylyi recA* deletion (RAM) recipient and DNA extracted from various wild-type integron-carrying species; vi) with the double mutant strain *A. baylyi* SD9 (Δ*recBCD* Δ*sbcCD*) as recipient; vii) and with competence mutant strain *A. baylyi* KOC4 (Δ*comFECB*) as a recipient. Selection of the transformants was done with different antibiotics and concentrations (see above in “bacterial strains and culture” and Table 1), according to the known or established resistance levels of the donors as determined by resistance typing and MIC determination.

A positive control was included to verify recipient cell competence and reproducible experimental conditions (transformation of *A. baylyi* BD413 by DNA from *A. baylyi* KTG which contains a chromosomally located *nptII* [kanamycin resistance] gene [47], [53]). As negative control recipient cells were streaked on LBR plus selective antibiotic without addition of the donor DNA. The transformation frequencies were calculated for each transformation assay and are given as the number of transformants divided by the number of viable recipient cells.

### Antimicrobial susceptibility tests

Antimicrobial susceptibility of donor, recipient and transformant bacteria was assessed by the disk diffusion method and determination of the minimal inhibitory concentrations (MICs). Both methods were performed according to the CLSI (Clinical Laboratory Standards Institute) guidelines [54], using Mueller-Hinton II (Fluka, BioChemika, Switzerland or Scharlau Chemie S.A., Spain) or PDM (AB Biodisk, Sweden) agar plates. The antibiotic susceptibilities tested for the various donor and transformant bacteria were determined according to the gene cassettes present. The antimicrobial disks used (Oxoid, England or AB Biodisk, Sweden) were: amikacin (30 µg), amoxicillin (10 µg), amoxicillin/clavulanic acid (20+10 µg), ampicillin (10 µg), cefotaxime (30 µg), ceftazidime (30 µg), chloramphenicol (5 µg), compound sulphonamides (300 µg), gentamicin (10 µg), imipenem (10 µg), kanamycin (30 µg), meropenem (10 µg), netilmicin (30 µg), rifampicin (5 µg), spectinomycin (100 µg), sulfadiazine (250 µg), streptomycin (10 µg), sulfamethoxazole/trimethoprim (25 µg), trimethoprim (5 µg), tobramycin (10 µg). When the antimicrobial susceptibility profile of transformants had changed, the E-test method (AB Biodisk, Sweden) was used to quantify the MICs. MICs were also determined prior to the experiments for both recipient and donor bacteria, to determine the appropriate concentration of antibiotics to be used for transformant selection. The following E-tests were used: ampicillin, cefotaxime, ceftazidime, gentamicin, kanamycin, spectinomycin, sulphamethoxazole, and tobramycin.

### PCR-based detection of integrons, gene cassettes, insertion sequences and other resistance determinants

The presence of class 1 integrons in the donor, recipient and transformant bacteria was assessed by PCR. PCR assays were set up in two different mixtures: 25 µl final volume of 22.5 µl PCR SuperMix (Invitrogen, Alfagene, Portugal), 0.75 µl of each primer 10 µM and 1 µl (approx. 10 ng DNA) of lysate DNA; or 50 µl final volume using 22.5 µl of the 2× PCR MasterMix Dynazyme II from Finnzymes (Finnzymes, Finland), 0.75 µl of each primer 10 µM, 25 µl of sterile water and 1 µl (approx. 10 ng DNA) of lysate DNA. PCR amplification was performed with a T-personal (Biometra, Göttingen, Germany), a MJ Mini (BIO-RAD, Portugal) or a PTC-200 (BIO-RAD, Norway) thermal cycler. Class 1 integrons were detected with a set of primers specific for the 5′-CS and the 3′-CS regions [24] or for the conserved regions of the class 1 integrase gene, *IntI1* [55]. The DNA amplification program consisted of an initial denaturation step (94°C, 5 min) followed by 35 cycles of denaturation (94°C, 1 min), annealing (55°C, 1 min) and extension (72°C, 5 min), and a single final extension of 16 min at 72°C for detection of integrons [28] or was performed for 30 cycles of denaturation at 94°C for 30 s, annealing at 65°C for 30 s and extension at 72°C for 45 s, followed by a final extension time of 10 min at 72°C [55], for amplification of the integrase gene.

Rearrangements of gene cassettes during integron transfer and transformant cultivation were tested by PCR combining one primer for the 5′-CS region and one primer that binds in the distal gene cassette in the integron of the donor bacterium.

For some transformants, obtained with strains SD2, KOI and RAM as recipients, the presence of the gene cassette *aadB* was determined by PCR with primers AADB1 (5′-ACGCAAGCACGATGATATTG-3′) and AADB2 (5′-CGCAAGACCTCAACCTTTTC-3′) for 5 min at 94°C, 30 cycles of 1 min at 94°C, 1 min at 55°C and 1.5 min at 72°C, followed by 10 min at 72°C.

Transformants with reduced susceptibility to some antibiotics but without a positive detection of the entire integron by PCR, were screened for the acquisition of different resistance determinants. PCRs were performed that targeted the gene cassettes present in the variable region of the integrons and the genes present in the 3′-CS of the integron of the corresponding donor bacteria, with specific primers for each cassette. The presence of the insertion sequence IS*Aba1* [56] was screened for by PCR in the recipient *A. baylyi*, in *A. baumannii* donors and in transformants with reduced susceptibility to ampicillin. For all PCR analyses, DNA extracted from the recipient and donor bacteria were used as negative and positive controls, respectively.

Transformants with reduced susceptibility to ampicillin, but that did not yield positive PCR products for the presence of integrons, were also tested for β-lactamase activity using a qualitative chromogenic method, with nitrocefin disks (AB Biodisk, Sweden) according to the manufacturer's instructions. The randomly selected transformants showed reduced susceptibility to ampicillin; donors and recipient bacteria were used as positive and negative controls for β-lactamase production.

### DNA sequencing

The genetic composition of the integrons of two of the donor bacteria, *A. baumannii* 064 and *S. enterica* serovar Rissen 486, as well as the flanking genomic regions of donor and transformant bacteria were determined by direct sequencing of genomic DNA and primer walking using the BigDye 3.1 cycle sequencing terminator reactions (Applied Biosystems) and an ABI3130XL genetic analyzer, as previously described [53], [57]. The composition of the integrons was determined by sequencing of the integron PCR-product with primers 5′-CS and 3′-CS and an additional pair of primers for *S. enterica* serovar Rissen 486, VS1 (5′-CTGGCTGCGTAGTTGTTTCA-3′) and VS2 (5′-GGGCTGCGAGTTCAATAG-3′). The first primers used in integron flanking regions, CS3 (5′-TCTCTACGACGATGATTTACACG-3′) and CS2 (5′-CGAATGGACAGCGAGGAG-3′), were designed based on the conserved regions sequence of class 1 integrons (accession number M73819) and the flanking region sequences were obtained by primer walking, using the software Primer3 (http://fokker.wi.mit.edu/primer3/input.htm) and Oligoanalyzer (http://www.idtdna.com/analyzer/Applications/OligoAnalyzer/). Sequences were edited and aligned in the Sequencher v.4.2.2 program (GeneCodes, USA) and identified using the BLASTN program (http://www.ncbi.nlm.nih.gov).

The genomes of transformants (St)3 and (AbII)3 (Table 1) were sequenced on the Roche/454 GS FLX Titanium platform, using one full plate and multiplexing of three 8 kb paired-end libraries. Between 137,080 and 283,658 sequence reads were generated per genome, resulting in single-scaffold assemblies with a length of 3,614,029 bp; and 3,667,429 bp and average sequencing depths of 13-fold, and 28-fold for (AbII)3, and (St)3, respectively. Sequence trimming, assembly, gene finding and annotation were performed with the automated CloVR-Microbe pipeline [58], [59], which is part of the Cloud Virtual Resource (CloVR) appliance [60] developed in the CloVR project (http://clovr.org). Briefly, raw sequence data were filtered and trimmed for quality and adaptor removal, and assembled with Celera Assembler [61]. Gene predictions and functional annotations were carried out using the tools and decision process described in the IGS Standard Operating Procedure for Automated Prokaryotic Annotation [62]. The annotated assemblies resulted in between two and 20 scaffolds, i.e. one or more contigs bridged by paired-end reads. In each case, only one scaffold was larger than 10,000 bp. None of the smaller contigs showed significant sequence similarity to plasmid and/or phage sequences and were considered assembly artifacts. Fasta and annotated Genbank files of all three assemblies are available from the authors.

### Pulse-field gel-electrophoresis (PFGE) and Southern blot hybridization

Genomic DNA was prepared in agarose blocks and digested for 3 h at 37°C with the endonuclease I-*Ceu*I (New England Biolabs, Beverly, MA) that specifically recognize the rRNA operons [63]. The I-*Ceu*I fragments were separated in a 1% agarose by PFGE using a CHEF-DR III apparatus (Bio-Rad, Hercules, Calif.) at 15°C, 6 V/cm with pulse time ramped from 20 s to 120 s over 11 h, followed by ramping from 60 s to 100 s for 11 h (adapted from Liu *et al.*, [63]). The separated DNA was transferred by vacuum blotting (Vacugene XL, Pharmacia Biotech) to a positively charged nylon membrane (Roche, Germany) as described by Sambrook *et al.* [64]. The 16S rRNA [65] and *intI1* [55] probes were amplified by PCR and labeled by using a PCR digoxigenin (DIG) probe synthesis kit (Roche Diagnostics, Basel, Switzerland). A DIG luminescent detection kit (Roche) was used according to the manufacturer's instructions. Co-hybridization for the 16S RNA and for the class 1 integrase *intI1* was performed at 68°C.

### Reverse-transcriptase PCR (RT-PCR)

Total RNA was isolated using NucleoSpin® Triprep (Macherey-Nagel, Germany) according to the manufacturer's instructions. Residual genomic DNA was removed by treatment with rDNase (Macherey-Nagel, Germany), followed by RNA ethanol precipitation. Reverse transcription was performed with 1 µg of RNA using the MonsterScript 1st-Strand cDNA synthesis kit with random 9-mer primers (Epicentre, USA), and the resulting cDNA was used in PCR reactions. The primers HS464 and HS463a [55] amplified a 473-bp fragment of *intI1*, and primers 16SF and 16SR [66] an approx. 1500-bp fragment of 16S rRNA. Isolated RNA was included in the PCR analyses to verify absence of DNA, and PCR targeting the 16S rRNA genes was used to confirm successful cDNA synthesis.

### Biological fitness measurements

The relative fitness (W) of integron-carrying transformants was estimated by pairwise competition experiments between transformants and the untransformed recipient strain in S2-minimal medium for 24 h, as previously described [53]. Individual transformants containing one out of five distinct integrons with the following resistance genes were evaluated: *bla*_IMP-5_ (transformant SD3); *bla*_OXA-30_ *+aadA1* (transformants [St]3, [SD1]1, SD4); *dfrA12*+*aadA2* (transformant SD5); *aacA4*+*bla_PSE_*+*aadA2* (transformant SD6); *aadB* (transformants SD2, [SD2]1). Selection was performed with CTX 20 µg/ml (transformant SD3), SC 10 µg/ml+AM 5 µg/ml (transformants [St]3, [SD1]1, SD4), SC 20 µg/ml+W 250 µg/ml (transformant SD5), SC 20 µg/ml+AM 50 µg/ml (transformant SD6), or with K 25 µg/ml (transformants SD2, [SD2]1). Nineteen to 24 competition replicates were done for each transformant. The relative fitness (W) was calculated as the ratio of the Malthusian parameter of each competitor.

## Results

### Location and composition of integrons in the donor bacteria

The recipient strain *A. baylyi* BD413 does not carry identifiable integrons [44] as also confirmed by our own results from PCR and genome and direct DNA sequencing of strain BD413. The availability of multidrug resistant and integron-containing isolates in our own strain collection determined the initial selection of strains used as a source of donor DNA in our investigation. In order to be able to determine the stability of the integrons from the donor genomes during transformation of *A. baylyi*, the composition and genomic context of all integrons in the donor genomes was assessed by integron-specific PCR, and DNA sequencing by primer walking. PCR with primer pairs specific for the amplification of integron gene cassettes yielded a single product, which in all cases, upon sequencing, contained at least one known antibiotic resistance gene. The direct sequencing of each PCR product indicated the presence of a single type of integron in each donor genome. In *A. baumannii* 064, the integron harbored a central region of 763 bp, including the *aadB* gene (CDS 597 bp), which is known to encode an aminoglycoside adenyltransferase, responsible for gentamicin, kanamycin and tobramycin resistance. The integron of *S. enterica* serovar Rissen 486 contained a central region of 1913 bp, with two gene cassettes: *dfrA12* (CDS 498 bp), and *aadA2*, (CDS 792 bp). The *dfrA12* gene encodes a dihydrofolate reductase that confers resistance to trimethoprim, and the *aadA2* gene encodes an aminoglycoside adenyltransferase, responsible for streptomycin and spectinomycin resistance.

The genomic location of the integrons in the donor genomes was determined by PFGE. Co-hybridization of the *intI1* and 16S rRNA probes was interpreted as indicating chromosomal location, whereas hybridization only with the *intI1* probe was interpreted as indicating a plasmid location of the integron. A plasmid location of the integron was shown in the donors *S. enterica* serovar Typhimurium 490, *E. cloacae* C2R371 and *C. freundii* C16R385, while integrons in the *A. baumannii* 064, *S. enterica* serovar Rissen 486, *E. coli* C10R379, *A. baumannii* 65FFC [28] and *P. aeruginosa* SM [67] were located on the chromosome. The *A. baumannii* 064 and *S. enterica* serovar Rissen 486 strains both contain a plasmid, as observed by agarose gel electrophoresis (data not shown). *E. fergusonii* AS041A2 also harbored a plasmid and repeated PFGE results were not conclusive for the determination of the location of the integron in this strain.

Sequencing of the flanking regions of the integrons revealed that their insertion sites varied in the donors, and that the integrons were often linked to transposable elements. In *S. enterica* serovar Typhimurium 490, the integron was inserted in an intact Tn*21*-like transposon [36]. The integron of *A. baumannii* 064 was inserted in a Tn*1721*-like transposon [68], [69], flanked by the insertion sequence IS*26* on both sides, forming an IS*26*-composite transposon. The *A. baumannii* 65FFC integron was embedded in a defective Tn*402*-like transposon, flanked by a 439 bp Miniature Inverted-repeat Transposable Element (MITE) on both sides [70].

In *S. enterica* serovar Rissen 486, the *tnpA*, *tnpR* and *tnpM* genes were detected next to the 5′-CS region of the integron. The *chrA* gene, which codes for a putative truncated chromate ion transporter, was found flanking the 3′-CS region. The closest homologue to the *tnp* genes was found in the transposon Tn*1721*, suggesting the integron in serovar Rissen 486 may be localized inside a transposon. However, for technical reasons, we were unable to obtain additional DNA sequence by primer walking.

In *P. aeruginosa* SM, *tnpR* and *tnpM* genes were identified adjacent to the 5′-CS flanking region of the integron. The genes flanking the 3′-CS region could not be determined. In this case, the *tnp* genes were 100% identical to the genes found in the Tn*5051*-like transposon; also in this case indicating that the integron is located in a transposon.

The flanking regions of the integrons of *C. freundii* C16R385, *E. cloacae* C2R371, *E. coli* C10R379 and *E. fergusonii* AS041A2 were not determined.

### Interspecies transfer of integrons/gene cassettes into wild-type *A. baylyi*

#### Phenotypic characterization of transformants

Natural transformation of *A. baylyi* BD413 by integron-containing DNA from the related species *A. baumannii* occurred at frequencies up to 1.6×10^−7^ transformants per recipient over a 24 h period. Natural transformation was also seen after exposure to integron-containing DNA of strains from the unrelated bacterial species *C. freundii*, *E. coli*, *E. cloacae*, *K. pneumoniae*, *P. aeruginosa*, *S. enterica* serovar Rissen, *and S. enterica* serovar Typhimurium, at frequencies ranging from 4.4×10^−9^ to 2.1×10^−7^ (Table 2). The only two integron-containing DNA sources tested and not capable of transforming wildtype *A. baylyi* BD413 above the detection limit of <1 transformant per 10^9^ bacteria were two *Escherichia* isolates (*E. coli* C10R379 and *E. fergusonii* AS041A2). In addition to purified DNA, the supernatant of a heat-killed bacterial cell suspension also gave rise to bacterial transformants (Table 2) at comparable frequencies. Thus, DNA purity seems to be of limited importance. As expected, natural transformation of the competence deficient mutant *A. baylyi* KOC4 as a recipient did not yield transformants.

**Table 2:** Natural transformation of *A. baylyi* with genomic DNA from different sources.

Recipient strain	Donor straina)	Mean no. of transformants (CFU) ± SD	Mean no. of recipients (CFU) ± SD	Transformants per recipientsb)
*A. baylyi* BD413	Purified DNA:			
	*A. baumannii* 064	(3.2±3.1)×10^1^	>(1.5±0.4)×10^9^	<2.1×10^−8^ c)
	*A. baumannii* 65FFC	(2.6±2.5)×10^2^	>(1.6±0.4)×10^9^	<1.6×10^−7^
	*C. freundii* C16R385	(5.1±4.0)×10^0^	(4.3±0.8)×10^8^	1.2×10^−8^
	*E. cloacae* C2R371	(5.1±2.3)×10^0^	(2.8±0.0)×10^8^	1.9×10^−8^
	*E. coli* C10R379	0	(7.9±4.4)×10^8^	0
	*E. coli* K71-77	(1.0±0.6)×10^2^	(4.7±0.9)×10^8^	2.1×10^−7^
	*E. fergusonii* AS041A2	0	>1.7×10^9^	0
	*K. pneumoniae* K66-45	(2.2±3.4)×10^0^	(4.9±1.0)×10^8^	4.4×10^−9^
	*P. aeruginosa* K34-73	(3.3±4.7)×10^1^	(4.8±0.7)×10^8^	7.0×10^−8^
	*P. aeruginosa* SM	(4.4±4.3)×10^1^	>(1.6±0.5)×10^9^	<2.8×10^−8^
	*S. enterica* Rissen 486	(3.1±3.0)×10^2^	>(1.7±0.2)×10^9^	<1.8×10^−7^
	*S. enterica* Thyp. 490	(1.4±1.3)×10^2^	>(1.4±0.6)×10^9^	<1×10^−7^ c)
	Supernatant/lysate with DNA:			
	*A. baumannii* 064	(1.7±0.1)×10^2^	>(1.9±0.4)×10^9^	<8.9×10^−8^
	*A. baumannii* 65FFC	(2.0±1,5)×10^2^	>(1.7±0)×10^9^	<1.2×10^−7^
	*P. aeruginosa* SM	(1.7±0.7)×10^2^	>(1.2±0.7)×10^9^	<1.4×10^−7^
	*S. enterica* Rissen 486	(3.6±0.2)×10^2^	>(1.7±0)×10^9^	<2.1×10^−7^
	*S. enterica* Thyp. 490	(3.6±0.9)×10^1^	(8.1±5.2)×10^9^	4.4×10^−8^
*A. baylyi* SD2	*A. baumannii* 65FFC	(1.6±0.3)×10^5^	(3.0±0.2)×10^8^	5.3×10^−4^
	*C. freundii* C16R385	(6.7±8.7)×10^4^	(9.1±1.1)×10^8^	7.4×10^−5^
	*E. cloacae* C2R371	(2.9±0.2)×10^4^	>(1.0±0.6)×10^9^	<2.9×10^−5^
	*E. coli* C10R379	(1.2±0.7)×10^4^	(9.9±1.4)×10^8^	1.2×10^−5^
	*E. fergusonii* AS041A2	(3.1±0.2)×10^4^	>(1.3±0.6)×10^9^	<2.4×10^−5^
	*P. aeruginosa* SM	(4.9±1.0)×10^3^	(5.2±0.9)×10^8^	9.4×10^−6^
	*S. enterica* Rissen 486	(9.4±3.9)×10^3^	(4.7±0.2)×10^8^	2.0×10^−5^
	*S. enterica* Thyp. 490	(2.0±0.4)×10^4^	(5.3±1.2)×10^8^	3.7×10^−5^
*A. baylyi* BD413	*A. baylyi* SD1	>3×10^5^	(6.6±0.5)×10^8^	>4.5×10^−4^
	*A. baylyi* SD2	(3.0±0.1)×10^5^	(5.8±0.3)×10^8^	5.2×10^−4^
	*A. baylyi* SD3	(5.5±2.8)×10^4^	>1.7×10^9^	<3.3×10^−5^
	*A. baylyi* SD4	(4.1±1.4)×10^3^	>1.7×10^9^	<2.5×10^−6^
	*A. baylyi* SD5	(1.9±0.4)×10^4^	>1.7×10^9^	<1.2×10^−5^
	*A. baylyi* SD6	(1.6±1.3)×10^5^	>1.7×10^9^	<9.6×10^−5^
*A. baylyi* KOI	*A. baumannii* 65FFC	(3.2±4.8)×10^5^	>(1.3±0.6)×10^9^	<2.5×10^−4^
	*P. aeruginosa* SM	(3.5±0.9)×10^4^	>1.7×10^9^	<2.1×10^−5^
	*S. enterica* Rissen 486	(2.7±0.2)×10^2^	>1.7×10^9^	<1.7×10^−7^
	*S. enterica* Thyp. 490	(7.3±4.4)×10^4^	>1.7×10^9^	<4.4×10^−5^
*A. baylyi* KOI	*A. baylyi* KOI (Ps)1	(2.9±0.3)×10^6^	>1.7×10^9^	<1.8×10^−3^
*A. baylyi* RAM	*A. baumannii* 65FFC	(5.4±2.0)×10^0^	>1.7×10^9^	<3.3×10^−9^
	*P. aeruginosa* SM	(7.5±7.6)×10^1^	>1.7×10^9^	<4.6×10^−10^
	*S. enterica* Rissen 486	(2.1±3.3)×10^0^	>1.7×10^9^	<1.3×10^−9^
	*S. enterica* Thyp. 490	(1.9±0.8)×10^0^	>1.7×10^9^	<1.1×10^−9^
*A. baylyi* SD9	*S. enterica* Thyp. 490	(2.0±1.5)×10^0^	(1.7±0.1)×10^8^	1.1×10^−8^

a) Natural transformation assays were performed with purified DNA as donor source, except in the experiments with the various donor species and *A. baylyi* BD413 recipient bacteria, where supernatants of heat-treated cell suspensions were used as the sole source of DNA.

b) The transformation frequency obtained with DNA from positive control source (*A. baylyi* KTG with the *ntpII* gene) was 4.7×10^−4^. Negative control experiments, consisting of the same experimental conditions, with the exception of that no DNA was added, were included in each transformation assay.

c) The transformation frequency is, when calculated as the frequency per exposed unit of DNA: 1.3×10^−8^ transformants per genome equivalent and 7.5×10^−8^ transformants per genome equivalent for *A. baumannii* 064 and *S. enterica* serovar Typhimurium 490, respectively. The genome equivalent size is based on the average size calculated from published genomes of the 2 species, as available in GenBank (Apr. 2012); 3.95 Mb and 4.99 Mb, respectively).

Antimicrobial susceptibility profiles were determined for selected transformants (Table S1). The susceptibility profiles of some of the transformants were altered in ways that could not be explained by the resistance phenotypes encoded by the transferred complete integron. The hypothetical transfer of single gene cassettes rather than the entire integron could have explained the reduced susceptibility of transformants if such cassettes would have been inserted next to a host region providing promoter functions. However, none of the resistance genes, present as part of the integrons of the donor bacteria, could be identified by gene-cassette specific PCR analyses in the tested atypical transformants.

Transfer of the insertion sequence IS*Aba1* could hypothetically explain reduced ampicillin susceptibility in some transformants, due to induction of expression of the *A. baylyi ampC* gene [56]. However, this insertion sequence was not detected by PCR in the transformants tested. The transfer of other β-lactamase determinants unrelated to the integron could also explain reduced ampicillin susceptibility in some transformants. Observed transfer of β-lactamase was detected in two transformants resulting from exposure to DNA of *A. baumannii* 064 DNA [(AbII)4 and (AbII)L1]; however, the molecular basis for the resistance was not determined. See footnote of table S1 for an explanation of the abbreviations of transformants.

#### Genotypic characterization of transformants

**Figure 1:**
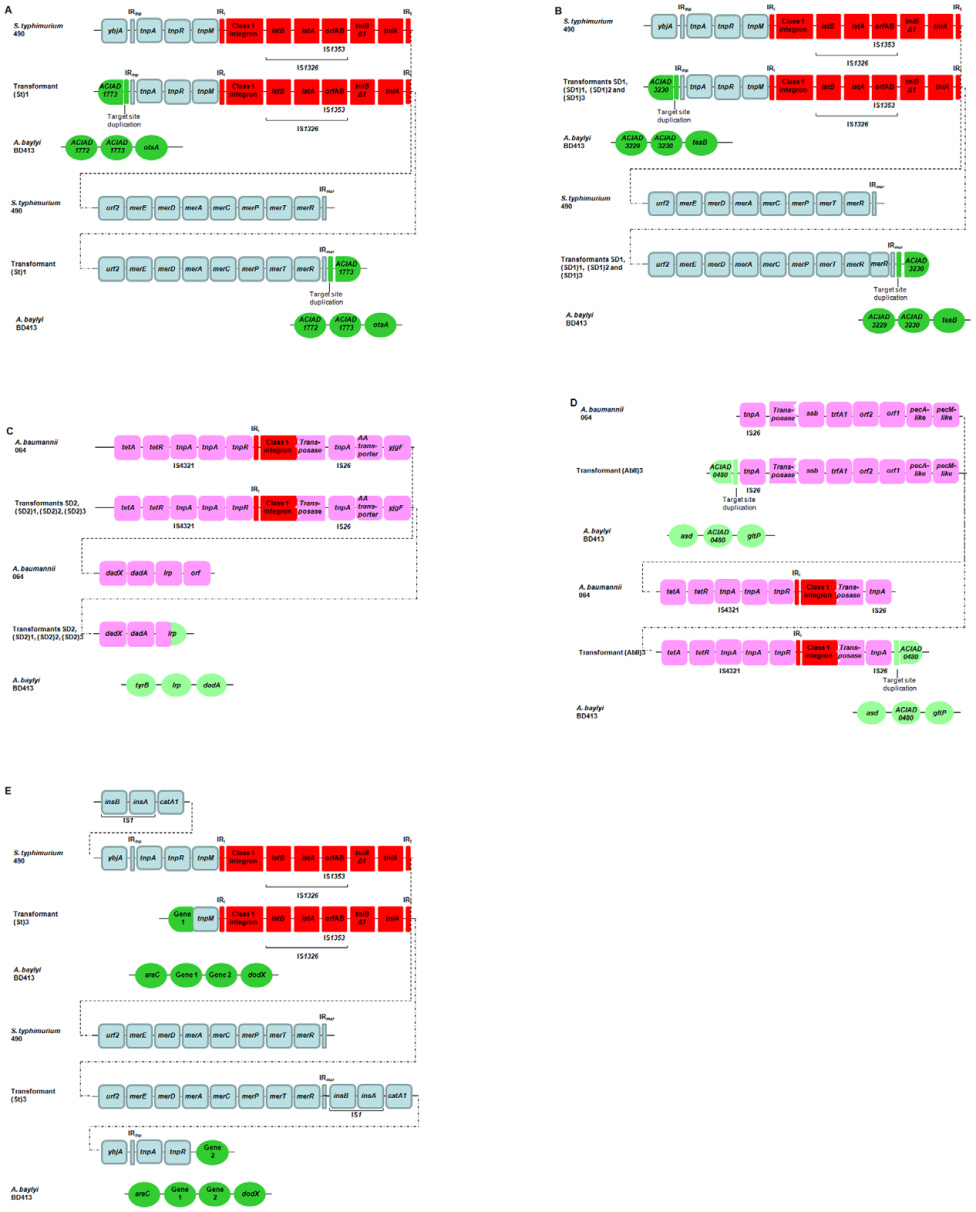
DNA fragments acquired from *S. enterica* and *A. baumannii* by the *A. baylyi* transformants. A) Transformant (St)1 obtained after exposure to DNA of *S. enterica* serovar Typhimurium 490. B) Transformants SD1, (SD1)1, (SD1)2 and (SD1)3 obtained after exposure to DNA of *S. enterica* serovar Typhimurium 490. C) Transformants SD2, (SD2)1, (SD2)2 and (SD2)3 obtained after exposure of DNA of *A. baumannii* 064, D) transformants (AbII)3 obtained after exposure to DNA of *A. baumannii* 064, E) transformant (St)3 obtained after exposure to DNA of *S. enterica* serovar Typhimurium 490; Gene 1 – gene coding for a bacterial regulatory, tetR family protein; Gene 2 – gene coding for a flavodoxin-like fold family protein. Class 1 integron includes the *intI1*, gene cassettes, *qacΔE*, *sulI1* and *orf5* genes.

Among all the phenotypes with increased resistance observed after exposure of the BD413 strain to DNA extracted from various integron-carrying species, four transformants were shown to have acquired the complete integron from *S. enterica* serovar Typhimurium 490 [named SD1, (St)1, (St)2 and (St)3], and another four transformants the entire integron from *A. baumannii* 064 [named SD2, (AbII)1, (AbII)2 and (AbII)3], as confirmed by PCR (Figure S1A) and DNA sequencing. The DNA sequence of the integron and integron flanking regions of the same transformants were determined. In all cases, acquisition of complete integrons plus additional flanking DNA was shown. Flanking acquired DNA fragments were up to 23,000 bp long and included in several cases transposable elements or other genes with important phenotypes, such as a chloramphenicol resistance gene (*catA2*). Figure 1 summarizes the genetic composition of the DNA acquired by the individual *A. baylyi* transformants.

In the transformants (St)1 and SD1, obtained after exposure to DNA of *S. enterica* serovar Typhimurium 490, the incorporated DNA region was found to consist a copy of transposon Tn*21*-like, which also contained the integron. In both cases, target site duplications of 4 and 5 bp, respectively, were identified providing evidence for that the (St)1 and SD1 transformants resulted from active transposition of Tn*21*-like (figure S2A). The transformant SD1 also showed a partial duplication of the *merR* gene of the transposon. In transformant (St)1, the integron-containing transposon had inserted into the *A. baylyi* gene ACIAD1773, which encodes a putative transport protein permease (Figure 1A). In transformant SD1, the integron-containing transposon had inserted into gene ACIAD3230, encoding a putative proton/sodium-glutamate symport protein (Figure 1B).

Whole-genome sequence analysis of *A. baylyi* transformant (St)3 revealed that the integron had transferred as part of a Tn*21*-like transposon. The incorporated DNA was found in the transformant genome in a region containing a 54 kb stretch of DNA not present in the published *A. baylyi* ADP1 strain. This DNA stretch has not been described before, and appears to be a prophage region, inserted in the ACIADtRNASer_34 gene. The transposon present in transformant (St)3 also showed rearrangement compared with its donor, *S. enterica* serovar Typhimurium 490. In (St)3, the *tnpR*, *tnpA*, *ybjA*, *catA1* and IS*1* genes were located at the 3′-CS region, flanking the *mer* operon, whereas in the previously described Tn*21* sequences, they are located in the 5′-CS region (Figure 1E). The rearrangement was confirmed by sequencing as well as by PCR.

DNA sequencing by primer walking also showed that transformant (St)2 acquired the integron embedded in the Tn*21*-like transposon, without rearrangements in this case, and the insertion had also occurred in the 54 kb region indicated above.

The *A. baylyi* transformant SD2, obtained after exposure to *A. baumannii* 064 DNA, also acquired considerable stretches of genomic donor DNA flanking the complete integron (Figure 1C). Extensive primer walking identified a crossover in the *lrp* gene of both the donor and recipient genomes, 5,428 bp from of the 3′-CS region of the integron. The second crossover junction was not identified but, based on primer walking results, had to be located at least at a distance of 10,500 bp from the 5′-CS region of the integron; the acquired DNA in this region included genes similar to the Tn*1721* transposon. The high nucleotide similarities between the *lrp* gene of the donor and the recipient and the characteristics of the crossover junctions suggest that *rec*A-dependent homologous (also called homeologous, heterologous, or heterogamic) recombination had facilitated the acquisition of the integron-containing DNA, a process recently investigated in detail for *A. baumannii* donor and *A. baylyi* recipient cells [53].

Whole genome sequencing of the *A. baylyi* transformant (AbII)3 revealed that it had acquired approximately 18,625 bps of DNA sequence from the *A. baumannii* 064 genome, which contained a IS*26*-composite transposon (Figure 1D); the horizontally acquired fragment was submitted to GenBank under the accession number JX041889. The insertion contained the integron embedded in a transposon-like region including a *tet* (tetracycline resistance) gene and a *tnpA* transposase gene (74% similar to the *tnp*A gene of Tn*1721*), and further flanked on both sides by the IS*26* elements. A target site duplication of 8 bp on both sides of the IS*26* insertion suggests transfer of the entire 18,625 bp segment occurred by transposition facilitated by IS26 sequences, into the ACIAD0480 gene of *A. baylyi* (encoding a putative membrane protein). Analysis of additional transformants, exposed to DNA of *A. baumannii* 064 by primer walking allied with PCR confirmed that the integrons acquired by transformants (AbII)1 and (AbII)2 were also located within a Tn*1721*-like transposon, and flanked by a IS*26* element. Although the exact locations in the *A. baylyi* genome were not determined for the latter, the obtained DNA sequence suggests a similar composition and mode of transfer as for transformant Ab(II)3.

The possible transfer of whole plasmids was investigated in the transformants collected after exposure to DNA from the plasmid-harboring *S. enterica* serovar Typhimurium 490 or *A. baumannii* 064. Although plasmids were clearly identified in both of the donor species by agarose gel-electrophoresis (data not shown) or PFGE, the same plasmids could not be detected in the transformants (Figure S3). The transformants SD1, (St)3, SD2 and (AbII)1 were shown by PFGE and probe hybridization to carry the acquired integron in the chromosome. It is noted that co-hybridization with the two probes targeting *intI*1 or the 16S rRNA genes did not take place for DNA extracted from transformants (St)1 and (St)2. Chromosomal location is nevertheless assumed because the larger fragment, where the integron of these two transformants is located, is not expected to hybridize with the 16S rRNA probe even though it is chromosomal. This is due to the opposite orientation of the rRNA operons at nucleotides 1660700–1666022 and 2941950–2947363 (access. number CR543861), leading to a 1.28 MB I-*Ceu*I fragment without the 16S rRNA gene. *A. baylyi* BD413 has seven rRNA operons, producing seven fragments after digestion with I-*Ceu*I [71], which can be seen for strain BD413 and transformants (St)1 and (St)2 (Figure S3A), lanes 2, 4 and 6, respectively. Variable locations, as well as different numbers, of the hybridizing band in the PFGE results of transformants (AbII)2 and (AbII)3 (Figure S3) led to several repetitions of the experiments; however both transformants repeatedly displayed a variable chromosomal location of the integrase sequence (data not shown), also confirmed by full genome sequence of one of these transformants, (AbII)3. This variability may hypothetically be due to gene amplification, which is reported to occur in *A. baylyi* [72], [73], followed by intra-genomic rearrangements. Rearrangements are often associated with repetitive sequences [74], which are known to represent 1.6% of the *A. baylyi* genome [44].

#### Effects of the RecBCD exonuclease

As several of the observed interspecies integron transfers appeared to have taken place through transposition, a transient existence of a linear double-stranded (ds) DNA intermediate in the cytoplasm was assumed. Such intermediates are thought to be substrates for the dsDNA-attacking exonuclease RecBCD [75], [76], and recent observations suggest that RecBCD also removes dsDNA-intermediates occurring during transformation (K. Harms et al., unpublished data). We deleted the *recBCD* operon (and the RecBCD suppressor genes *sbcCD* to obtain a wildtype-like viability; [49]) in the strain BD413, yielding strain SD9 which was employed as recipient in transformation experiments. Three of the SD9 transformants showed acquisition of integron from the *S. enterica* serovar Typhimurium 490. The low transformation frequencies obtained with this strain do not suggest that lack of RecBCD (and/or SbcCD) protects transformable cells from transposon activity initiated by linear double-stranded DNA present in the cytoplasm.

### Intraspecies transfer of integrons/gene cassettes in *A. baylyi*

To investigate whether acquisition of DNA in a first transformation would impact transformation efficiencies in subsequent experiments, transformation of *A. baylyi* BD413 by DNA extracted from *A. baylyi* transformants that previously acquired an integron (SD1, SD2, SD3, SD4, SD5 and SD6) was performed. Intraspecies transformation was 10- to 1,000-fold more efficient than interspecies transfer of the same integrons (Table 2).

Crossover junctions in the *A. baylyi* genome sequence were detected in 6 out of 6 tested transformants, suggesting that the observed intraspecies gene transfer occurred via homologous recombination. Transformant isolates (SD1)1, (SD1)2 and (SD1)3, obtained with the donor strain *A. baylyi* SD1, acquired at least 23,500 bp of the donor DNA. Transformant isolates (SD2)1, (SD2)2 and (SD2)3, obtained with the donor *A. baylyi* SD2, obtained at least 20,000 bp of the donor DNA. The exact size of the recombining regions for these isolates remains undetermined as the donor and recipient genomes have regions with identical DNA composition in the areas flanking the DNA insertion. Extensive DNA sequencing by primer walking of 6 transformants suggested that the composition of the donor DNA segments acquired in the initial interspecies transformation assay were maintained after subsequent intraspecies transformation (Figure 1A and C).

All of the tested transformants showed expected antimicrobial susceptibility profiles (Table S1) and had acquired the complete integron, which was confirmed by PCR (Figure S1C). The conserved composition of the acquired integron and the flanking regions suggest homologous recombination facilitated DNA exchange between the two *A. baylyi* genomes involved.

### Interspecies transfer of integrons/gene cassettes into integron-containing *A. baylyi*

To determine the impact of integrons resident in the recipient bacteria on the overall transformation efficiencies, we exposed integron-containing bacteria to chromosomal DNA with integrons of different compositions. Interspecies transformation of integron-containing *A. baylyi* recipient cells was 10- to 100-fold more efficient than interspecies transformation of wildtype *A. baylyi* cells (Table 2). The presence of integron sequences in the recipient cells (*A. baylyi* SD2) led to efficient replacement of gene cassettes rather than to the accumulation of additional integrons or gene cassettes in all tested transformants (76 out of 76) (figure S2B). Replacement was shown by changes in antimicrobial susceptibility profiles (Table S1) and the obtained fragment sizes by integron-specific PCR assays (Figure S1B). Recipient strains with integron-encoded *aadB* (SD2, KOI, RAM) lost the corresponding aminoglycoside resistance profiles upon transformation by genomic DNA containing different integrons (Table S1). Gene loss and substitution of the entire integron in the transformant strain was confirmed by PCR (Figure S1B). Rearrangements of gene cassettes within the integrons were not observed among the analyzed transformants. The expression of the acquired integrase gene in *A. baylyi* transformant SD2 was not detected in RT-PCR analyses (Figure S4), which might explain the absence of observable recombination of gene cassettes among the limited numbers of transformants examined.

Transformation frequencies of the *recA* mutant RAM were between 10^4^- to 10^5^-fold lower when compared to the *recA*-proficient recipient strain SD2 (Table 2), suggesting homologous recombination as mechanism responsible for efficient substitutive recombination.

#### Absence of effects of the integron-encoded integrase

To investigate whether the integrase was required for integron-acquisition or affected transformation efficiencies, experiments were repeated using the *intI1*::*cat*-inactivated integron-carrying *A. baylyi* strain KOI as a recipient. The resulting transformation frequencies were similar to those obtained with the recipient (SD2) harboring a functional integrase gene (Table 2). Of the 73 analyzed transformants, 70 acquired the entire gene cassette composition from the integron of the donor bacteria (Figure S1D). Further nucleotide analysis of the *intI1* region of these transformants revealed that the inactivated *intI1* region had been replaced with a functional copy from the donor bacteria in 4 out of 10 transformants after exposure to DNA from *A. baumannii* 65FFC, in 17 out of 21 from *P. aeruginosa* SM, in 10 out of 21 from *S. enterica* Rissen 486 and in 14 out of 21 from *S. enterica* Typhimurium 490. The remaining integrons maintained the inactive integrase of the recipient. The lack of an effect of the *intI1*-encoded integrase in the recombination process was also seen when the donor DNA contained an inactive integrase (Table 2); thus excluding a role of the integrase inter- and intraspecies acquisition of integrons or gene cassettes in *A. baylyi* recipients already containing an integron.

### Relative fitness assays

The biological cost of the acquired, integron-mediated, antibiotic resistance was determined for transformants SD3 (*bla*_IMP-5_), (St)3, (SD1)1 and SD4 (*bla*_OXA-30_ *+aadA1*), SD5 (*dfrA12*+*aadA2*), SD6 (*aacA4*+*bla_PSE_*+*aadA2*), *and* SD2 and (SD2)1 (*aadB*). The mean relative fitness (w) of these integron-carrying strains ranged from 0.96 to 1.01 (n = 19 to 24 replicates); only transformant SD3 showed a clear negative fitness effect, with w = 0.91 (n = 23; p = 0.001). In general, no consistent major differences in fitness were observed, suggesting that the horizontal acquisitions of integrons (including the co-transferred and often extensive additional DNA regions) do not lead to immediate and severe growth inhibition of transformant cells.

## Discussion

Horizontal gene transfer (HGT) is a key driver of bacterial adaptation and evolution [77]–[79]. A number of studies demonstrate that a significant amount of bacterial genomes are affected by HGT events [80]–[83]. In this study we examined to what extent DNA substrates and natural transformation can lead to interspecies transfer of distinct genetic elements encoding site-specific recombination mechanisms.

Annealing of complementary DNA strands in the cytoplasm is considered necessary for recircularization and hence stable uptake of plasmids in competent bacterial species [84] (K. Harms, unpublished data). Drawing on this general observation, we hypothesized that annealing of other single-stranded DNA taken up into the cytoplasm of competent bacterial cells would allow transient expression of genes present on such linear fragments. For instance, expression of recombinase genes present in mobile genetic elements in these double-strand DNA fragments could lead to integration of the elements into the host chromosome. To what extent natural transformation of bacteria can facilitate inter-genomic mobility of genetic elements being part of species-foreign, linear DNA fragments can be tested.

Indeed, we provide experimental data demonstrating that natural transformation can facilitate interspecies transfer of integrons and transposons that is not limited by the genetic relatedness of the donor with the host. Exposure of *A. baylyi* to DNA of an integron-containing *Salmonella* strain led to horizontal transfer of its integron due to transposition of a Tn*21*-like transposon that contained the integron. The Tn*21*-like transposon was found as single inserts at different chromosomal locations in 3 different examined transformant genomes. Exposure of *A. baylyi* to DNA of an integron containing *A. baumannii* strain also led to transformants with single chromosomal integrations of the integron. The integrations occurred by either DNA sequence similarity-based homologous recombination, or by genetic linkage to and movement of IS*26* elements. Resistance profiling, PCR-based target amplification and extensive DNA sequencing confirmed the acquisition and chromosomal insertion of complete integrons and its flanking DNA by *A. baylyi* transformant cells. The general acquisition process involved is illustrated in Figure S2A.

### Interspecies transfer into integron free *A. baylyi* cells

Purified DNA substrates of *A. baumannii* 064 and *S. enterica* serovar Typhimurium 490 transformed *A. baylyi* cells at frequencies of 10^−8^ and 10^−7^ over a 24 h time period. In all examined cases, the composition and order of gene cassettes in the donor bacteria was maintained in the transformant cells. Also DNA lysates could transform *A. baylyi* cells suggesting variability in DNA purity is of minor importance for HGT, as observed previously [51].

DNA sequencing of the flanking regions of the integrons in 6 initial transformants obtained after exposure to species-divergent donor DNA verified their incorporation into the *A. baylyi* BD413 chromosome. The genomic locations of the integrons were also maintained in subsequent transformants (n = 6) obtained after exposure to DNA isolated from the initial transformants. Sequencing of the flanking regions of the transferred integrons also revealed that not only the integron was acquired from the donor bacteria, but also additional DNA flanking the integrons. The length of the acquired, continual DNA fragments could be up to 23,000 bps long. The sequencing of the acquired DNA in 6 transformants obtained after exposure to DNA of various species, as well as Southern hybridization of a total of 8 transformants, revealed that integration had occurred in different regions of the *A. baylyi* genome.

Most interestingly, stable integration of the integron containing DNA in the *A. baylyi* chromosome had occurred by several mechanisms, depending of the flanking regions of the integrons. Transposition-based insertions were observed for integrons acquired from unrelated species. Specifically, DNA sequencing revealed that exposure to DNA substrates of *S. enterica* serovar Typhimurium 490 resulted in chromosomal integration of the acquired DNA due to the activity of transposase genes flanking the integrons; such as transposon Tn*21*-like. Integrons are often embedded in transposon Tn*21* [36], and many different gene cassette arrays have been reported for integrons present in Tn*21*-like transposons [85]. The horizontal dissemination of this transposon is a major contributor to the dissemination of integrons. Here we have experimentally demonstrated that natural transformation can facilitate interspecies movement of this transposon. For a related species (*A. baumannii* DNA donor), homologous recombination-based insertions were observed, as expected [53]. However, in this latter exposure scenario, also site-specific recombination due to IS*26*-associated transposition was observed.

Similarly to Tn*21*-like, IS*26* elements are often associated with antibiotic resistance genes and presumed active in *Acinetobacter* [86]. There are several reports of IS*26*-associated integrons [87], [88], and transposition of IS*26*-composite transposons has been suggested [89]. The presence in the recipient of nonfunctional IS*26* could also allow the capture of additional resistance genes by homologous recombination-mediated DNA integration, if a homologous IS*26* region is present in the donor DNA fragments. Such recombination scenario could explain, for example, the dissemination of the chromosomally-located integron-IS*26* in *A. baumannii* in South Korea [88], [90]. A previous bioinformatics-based study of Partridge et al. [41] also indicated transposition as a possible mechanism of integron movement. However, no experimental tests were performed.

The biological properties of natural transformation as a mechanism that can facilitate DNA translocation over bacterial membranes and site-specific recombination (e.g. active transposition events of transposons) in bacterial genomes could be further utilized. For instance, both model and applied systems can be developed using small DNA fragments with site-specific recombination functions as donor DNAs. Moreover, possible interactions between recombinases encoded by the incoming DNA and host enzymes should be investigated. For example, the *A. baylyi* ADP1 strain harbors two 2 prophages, a number of transposases, and 6 copies of IS*1236*, with two of them flanking Tn*5613* [44].

The exposure of *A. baylyi* cells to the various DNA substrates resulted in surprisingly high transformation frequencies, as determined by phenotypic screening on antibiotic-containing growth media. However, subsequent genotypic screening (PCR) showed that only some of the transformants with changed antibiotic resistance profiles had acquired entire integrons. The observation of variable changes in susceptibility patterns, after exposure to various sources of bacterial DNA, suggest the broad potential of and the presence of a yet not described range of transferable resistance determinants in bacteria, of unknown mechanistic and genetic nature. Our study suggests that exposure of competent bacteria to heterologous sources of DNA may produce complex changes in resistance profiles, not necessarily predictable from the known resistance genes present in a given donor isolate. In two examined cases, changes in resistance profile were due to the acquisition of β-lactamases as determined by the nitrocefin test. The broader effects of the composition of the *A. baylyi* genome after the heterologous recombination with DNA from the related species *A. baumannii* will be determined in a separate study. A recent study by Mell *et al.* [91] reported transfer of numerous chromosomal polymorphisms between *Haemophilus influenza* genomes after natural transformation.

### Inter- and intraspecies transfer into integron-containing *A. baylyi cells*

Natural transformation of the integron-carrying *A. baylyi* SD2 recipient with DNA isolated from different species of donor bacteria gave comparable transformation frequencies to those obtained with DNA of the same species. Thus, interspecies acquisitions of integrons that take place at low initial frequencies can be followed by high-frequency interspecies dissemination. Interestingly, all tested transformants acquired the gene cassette composition of the integron of the donor bacterium, rather than acquiring additional new gene cassettes in the existing integron. This result suggests that homologous recombination replaces gene cassettes through gene replacement caused by crossover junctions forming at the conserved end segments of the integron (Figure S2B). A role of the *intI1* integrase was not observed in our studies.

Transformation experiments performed with recipient cells containing an integron with the integrase gene inactivated (strain KOI), and also with recipient cells containing an integron, but with the *recA* gene inactivated (strain RAM) that resulted four orders of magnitude reduction in transformation frequencies, confirm the role of homologous recombination [53], [92] in the replacement of integrons and gene cassettes in these recipient bacteria. Partridge et al [41] also suggested replacement of a gene cassette array in *Pseudomonas aeruginosa* to have occurred by homologous recombination. Here we provide experimental evidence that homologous recombination can efficiently replace gene cassette arrays. Mobile genetic elements thus provide sufficient DNA similarity for homologous recombination to occur between otherwise unrelated bacterial species. The presence of DNA similarity can therefore likely contribute to the formation and spread of chromosomally-located complex mosaic regions, often formed by several resistance genes and mobile elements [93].

Recent publications demonstrate that many integrase genes in integrons carry LexA binding sites in the vicinity of their promoter regions and can be controlled by the host LexA protein (transcriptional repressor of the SOS response [94], [95]). In organisms harboring *lexA* alleles, SOS induction can lead to increased integrase activity resulting in increased frequencies of cassette rearrangements [94], [95]. SOS induction of the expression of the integrase by both conjugation [95] and natural transformation [96] has been recently shown in *V. cholerae*. In contrast, and unlike many Enterobacteriaceae, the SOS response in *A. baylyi* is not regulated by a LexA orthologue [97]. The integrase gene in our recipient strain is therefore derepressed or regulated by other unknown factors, as expression was not found in the RT-PCR.

The absence of detected rearrangements of gene cassettes in our study might be explained by the absence (or low) expression of the integrase in the recipient *A. baylyi* SD2, and by the fact that the homologous recombination events will occur much more frequently than integrase-catalyzed cassette recombination events. Therefore, rare rearrangements of gene cassettes may have occurred in our model system but would not be detectable in the limited number of transformants that were genetically characterized. The recent work published by Baharoglu et al. [95] on conjugative induction of SOS and its ability to trigger cassette recombination showed that cassette rearrangements occurred at very low frequencies (e.g. 10^−8^) in *V. cholerae*). The relative low frequency of integrase-facilitated cassettes recruitment and rearrangements, compared with the frequency of homologous recombination, can also help explain why some gene cassette arrays are common and shared between different bacterial species [98].

### General considerations

Intervention policies aimed at combating antibiotic resistance often rely on the assumption that resistance genes are costly and will be lost from pathogenic bacterial populations when antibiotic pressure is removed [99]. Our relative fitness measurements suggest that the acquired integrons and the resulting changes in resistance profiles did not cause major fitness reductions of transformants. It is noted that the fitness measurements represent the combined effect of all transferred DNA segments, as well as effects on the transformant genome due to insertion effects.

Natural competence is described in at least 60 species of bacteria, including several species present in clinical environments [100]. However, the proportion of species able to acquire DNA by natural transformation remains to be clarified, as many species or strains may have the capacity without such being detectable under laboratory conditions that are limited in sensitivity, time and by environmental variables [100]. The occurrence of naturally transformable species within the pool of uncultivable bacteria also remains to be fully explored. Interestingly, several species or strains, including *E. coli*, *A. baumannii* and *Pseudomonas stutzeri*, regarded as non-competent have later been shown to be naturally transformable under certain circumstances [101]–[104]. Natural transformation could, for instance, be responsible for the dissemination of chromosomal integrons in *P. aeruginosa* [105]. Recently, the acquisition of (synthetic) gene cassettes by natural transformation in a *Pseudomonas* species [39] was also shown; indicating that homology-independent DNA acquisitions might be more general phenomenon. It is also worth noting that chromosomal super-integrons are mainly found in *Vibrio* species, which are naturally transformable [9], [100].

Our results indicate that natural transformation of DNA fragments is not necessarily limited by requirements of high DNA sequence similarity for stable integration to occur; and that genetically unrelated bacteria can exchange genetic material (such as integron-containing transposons) through natural transformation provided the transferred DNA fragments encodes functions for site-specific recombination. As observed in our studies, the genetic signature of events of natural transformation can be limited to the site-specific insertions of transposons. However, the observation of transposons or insertion sequences in a given bacterial genome is usually not causally attributed to events of natural transformation. Thus, retrospective DNA sequence analysis would not have considered natural transformation as the causal mechanism until now.

This study suggests a significant potential for the interspecies spread of mobile genetic elements such as transposons, and insertion sequences through natural transformation. Hence, presenting a new pathway for horizontal dissemination of antimicrobial resistance determinants present in integrons embedded in transposons.

### Gene reference numbers

The reference numbers for genes mentioned in the text include: ACIAD0480 (2881081), ACIAD1773 (2878997), ACIAD3230 (2879415), ACIADtRNASer_34 (2877868) *aadA2* 486S (5741162), *ampC* (2880712), *dfrA12* (5741160), *intI1* (11934322) *recA* (2879476), *recB* (2879477), *recC* (2879478), *recD* (2879479), *lrp* (2878604), *sbcC* (2879936), *sbcD* (2879937), 16S rRNA (2880271) GeneID and *bla*_IMP-5_ (AF290912), *bla*_OXA-30_+*aadA1* (AY534545), *aacA4*+*bla*_PSE_+*aadA2* (DQ219465), *bla*_VIM-4_+*arr-7*+*aacA4*+*bla*_PSE-1_ (FN397623), IS*Aba1* (AY758396), MITE (JF810083), Tn*21*-like (AM991977) and Tn*5051*-like (AJ867812) accession number from the NCBI GenBank database. The IS*26*-composite transposon was submitted to GenBank with the accession number JX041889.

## Supporting Information

Figure S1 **Agarose gel electrophoresis of class 1 integron PCR-products.** A) Lane 1 – *A. baumannii* 064 (donor strain); 2–5 – transformants from *A. baumannii* 064, 4.10.1, 4.31.1, 4.34.1 and 4.46.1, respectively; 6 – *S. enterica* serovar Typhimurium 490 (donor strain); 7–10 – transformants from *S. enterica* serovar Typhimurium 490, 2.1.1, 2.3.6, 2.24.5 and 2.26.1, respectively; 11 – *A. baylyi* BD413 (recipient strain); 12- 1 Kb Plus DNA ladder (Invitrogen). B) Lane 1 – 1 Kb Plus DNA ladder (Invitrogen); 2 – *A. baumannii* 65FFC (donor strain); 3–5 – transformants from *A. baumannii* 65FFC, SD3, [SD2](AbI)1 and [SD2](AbI)2, respectively; 6 – *C. freundii* C16R385; 7–9 – transformants from *C. freundii* C16R385, [SD2](Cf)1, [SD2](Cf)2, [SD2](Cf)3, respectively; 10 – *E. cloacae* C2R371; 11–13 – transformants from *E. cloacae* C2R371, [SD2](Ecl)1, [SD2](Ecl)2, [SD2](Ecl)3, respectively; 14 – *E. coli* C10R379; 15–17 – transformants from *E. coli* C10R379, [SD2](Ec)1, [SD2](Ec)2, [SD2](Ec)3, respectively; 18 – transformant SD2 (recipient bacterium); 19 – *A. baylyi* BD413 (negative control); 20 – 1 Kb Plus DNA ladder (Invitrogen); 21 – *E. fergusonii* AS041A2; 22–24 – transformants from *E. fergusonii* AS041A2, [SD2](Ef)1, [SD2](Ef)2, [SD2](Ef)3, respectively; 25 – *P. aeruginosa* SM (donor strain); 26–28 – transformants from *P. aeruginosa* SM, SD6, [SD2](Ps)1, [SD2](Ps)2, respectively; 29 – *S. enterica* serovar Rissen 486 (donor strain); 30–32 – transformants from *S. enterica* serovar Rissen 486, SD5, [SD2](Sr)1, [SD2](Sr)2, respectively; 33 – *S. enterica* serovar Typhimurium 490 (donor strain); 34–36 – transformants from *S. enterica* serovar Typhimurium 490, SD4, [SD2](St)1, [SD2](St)2, respectively; 37 – transformant SD2 (recipient bacterium); 38 – *A. baylyi* BD413 (negative control). C) Lane 1 – transformant SD1 (donor strain); 2–4 – transformants from SD1, (SD1)1, (SD1)2, (SD1)3, respectively; 5 – transformant SD2 (donor strain); 6–8 – transformants from SD2, (SD2)1, (SD2)2, (SD2)3, respectively; 9 – transformant SD3; 10–12 – transformants from SD3, (SD3)1, (SD3)2, (SD3)3, respectively; 13 – transformant SD4; 14–16 - transformants from SD4, (SD4)1, (SD4)2, (SD4)3, respectively; 17 – transformant SD5; 18–20 – transformants from SD5, (SD5)1, (SD5)2, (SD5)3, respectively; 21 – transformant SD6; 22–24 – transformants from SD6, (SD6)1, (SD6)2, (SD6)3, respectively; 25 – *A. baylyi* BD413 (recipient strain); 26 – 1 Kb Plus DNA ladder (Invitrogen). D) Lane 1 – 1 Kb Plus DNA ladder (Invitrogen); 2 – *A. baumannii* 65FFC (donor strain); 3–5 – transformants from *A. baumannii* 65FFC, [KOI](AbI)1, [KOI](AbI)2, [KOI](AbI)3, respectively; 6 – *P. aeruginosa* SM (donor strain); 7–9 – transformants from *P. aeruginosa* SM, [KOI](Ps)1, [KOI](Ps)2, [KOI](Ps)3, respectively; 10 – *S. enterica* serovar Rissen 486 (donor strain); 11–13 – transformants from *S. enterica* serovar Rissen 486, [KOI](Sr)1, [KOI](Sr)2, [KOI](Sr)3, respectively; 14 – *S. enterica* serovar Typhimurium 490 (donor strain); 15–17 – transformants from *S. enterica* serovar Typhimurium 490, [KOI](St)1, [KOI](St)2, [KOI](St)3, respectively; 18 – transformant KOI (recipient bacterium); 19 – *A. baylyi* BD413 (negative control).
(TIF)

Figure S2 **Schematic presentation of the horizontal acquisitions.** A) Acquisition of a transposon by transposition with duplication of sequences around insertion site; B) Acquisition or substitution of gene cassettes by homologous recombination occurring between conserved regions of a class 1 integron.
(TIF)

Figure S3 **PFGE and Southern blot hybridization.** A) Pulse-field gelelectrophoresis (PFGE) of I-*Ceu*I-digested total DNA of recipient, donor and transformant bacteria; B) corresponding Southern hybridization with 16S rRNA probe; C) corresponding Southern hybridization with *intI*1 probe. Lane 1 – lambda PFG marker (New England Biolabs); 2 – *A. baylyi* BD413 (recipient); 3 – *S. enterica* serovar Typhimurium 490 (donor); 4–7 – transformants from exposure to DNA of *S. enterica* serovar Typhimurium 490, (St)1, SD1, (St)2, (St)3, respectively; 8 – *A. baumannii* 064 (donor); 9–12 – transformants from exposure to DNA of *A. baumannii* 064, SD2, (AbII)1, (AbII)2 and (AbII)3, respectively; 13 – *S. enterica* serovar Rissen 486 (donor); 14 – *E. cloacae* C2R371 (donor); 15 – *E. coli* C10R379 (donor); 16 – *C. freundii* C16R385 (donor); 17 – *E. fergusonii* AS041A2 (donor); 18 – lambda PFG marker (New England Biolabs).
(TIF)

Figure S4 **RT-PCR of the class 1 integrase RNA in transformant** ***A. baylyi*** **SD2.** Lane 1 (left) – SmartLadder (Eurogentec); 2–3 –PCR targeting the *intI1* gene, 2 – cDNA; 3 – RNA; 4–5 – PCR targeting the 16S rRNA gene, 4 – cDNA; 5 – RNA. For primers description, see Material and Methods.
(TIF)

Table S1 **Antimicrobial susceptibility of** ***A. baylyi*** **transformants determined by the E-test method.**
(DOC)
