# Supplementary figures and images for: Natural Transformation Facilitates Transfer of Transposons, Integrons and Gene Cassettes between Bacterial Species
Source: PLoS Pathog. 2012 Aug 2;8(8):e1002837. doi: 10.1371/journal.ppat.1002837 (PMC3410848; doi:10.1371/journal.ppat.1002837)

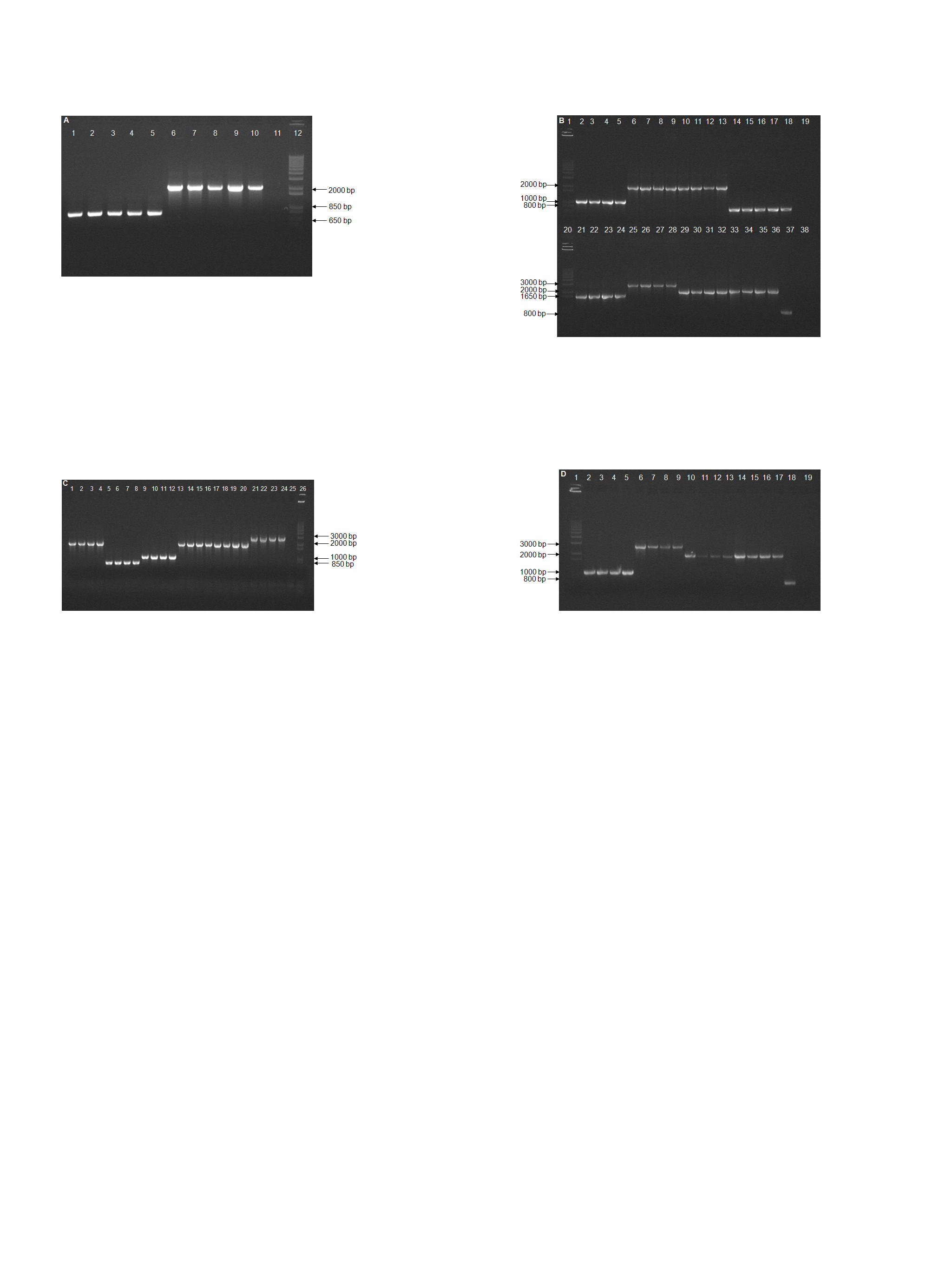

Supplement: Figure S1 — Agarose gel electrophoresis of class 1 integron PCR-products. A) Lane 1 – A. baumannii 064 (donor strain); 2–5 – transformants from A. baumannii 064, 4.10.1, 4.31.1, 4.34.1 and 4.46.1, respectively; 6 – S. enterica serovar Typhimurium 490 (donor strain); 7–10 – transformants from S. enterica serovar Typhimurium 490, 2.1.1, 2.3.6, 2.24.5 and 2.26.1, respectively; 11 – A. baylyi BD413 (recipient strain); 12- 1 Kb Plus DNA ladder (Invitrogen). B) Lane 1 – 1 Kb Plus DNA ladder (Invitrogen); 2 – A. baumannii 65FFC (donor strain); 3–5 – transformants from A. baumannii 65FFC, SD3, [SD2](AbI)1 and [SD2](AbI)2, respectively; 6 – C. freundii C16R385; 7–9 – transformants from C. freundii C16R385, [SD2](Cf)1, [SD2](Cf)2, [SD2](Cf)3, respectively; 10 – E. cloacae C2R371; 11–13 – transformants from E. cloacae C2R371, [SD2](Ecl)1, [SD2](Ecl)2, [SD2](Ecl)3, respectively; 14 – E. coli C10R379; 15–17 – transformants from E. coli C10R379, [SD2](Ec)1, [SD2](Ec)2, [SD2](Ec)3, respectively; 18 – transformant SD2 (recipient bacterium); 19 – A. baylyi BD413 (negative control); 20 – 1 Kb Plus DNA ladder (Invitrogen); 21 – E. fergusonii AS041A2; 22–24 – transformants from E. fergusonii AS041A2, [SD2](Ef)1, [SD2](Ef)2, [SD2](Ef)3, respectively; 25 – P. aeruginosa SM (donor strain); 26–28 – transformants from P. aeruginosa SM, SD6, [SD2](Ps)1, [SD2](Ps)2, respectively; 29 – S. enterica serovar Rissen 486 (donor strain); 30–32 – transformants from S. enterica serovar Rissen 486, SD5, [SD2](Sr)1, [SD2](Sr)2, respectively; 33 – S. enterica serovar Typhimurium 490 (donor strain); 34–36 – transformants from S. enterica serovar Typhimurium 490, SD4, [SD2](St)1, [SD2](St)2, respectively; 37 – transformant SD2 (recipient bacterium); 38 – A. baylyi BD413 (negative control). C) Lane 1 – transformant SD1 (donor strain); 2–4 – transformants from SD1, (SD1)1, (SD1)2, (SD1)3, respectively; 5 – transformant SD2 (donor strain); 6–8 – transformants from SD2, (SD2)1, (SD2)2, (SD2)3, respectively; 9 – transforma [file ppat.1002837.s001.tif]

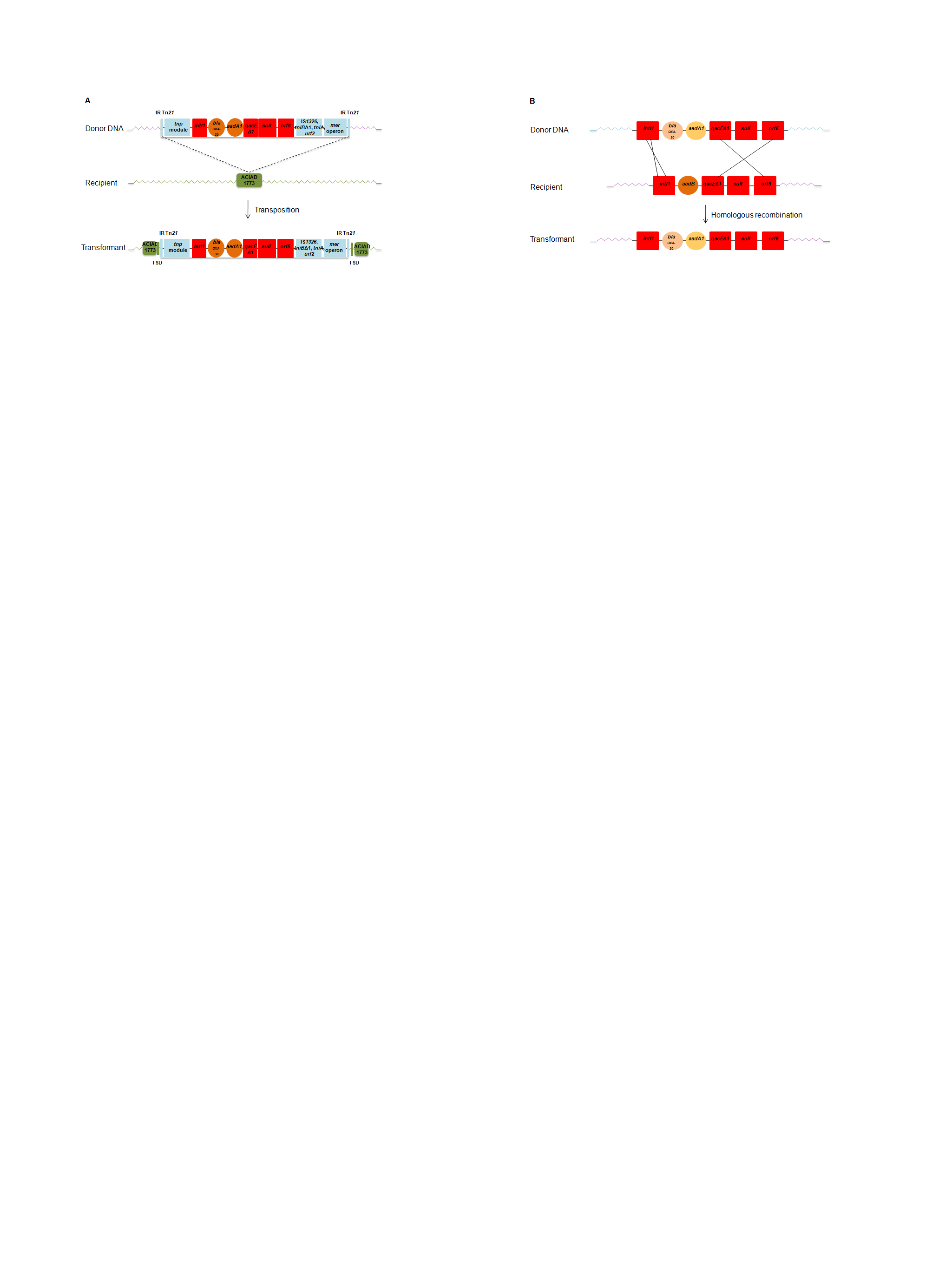

Supplement: Figure S2 — Schematic presentation of the horizontal acquisitions. A) Acquisition of a transposon by transposition with duplication of sequences around insertion site; B) Acquisition or substitution of gene cassettes by homologous recombination occurring between conserved regions of a class 1 integron. (TIF) [file ppat.1002837.s002.tif]

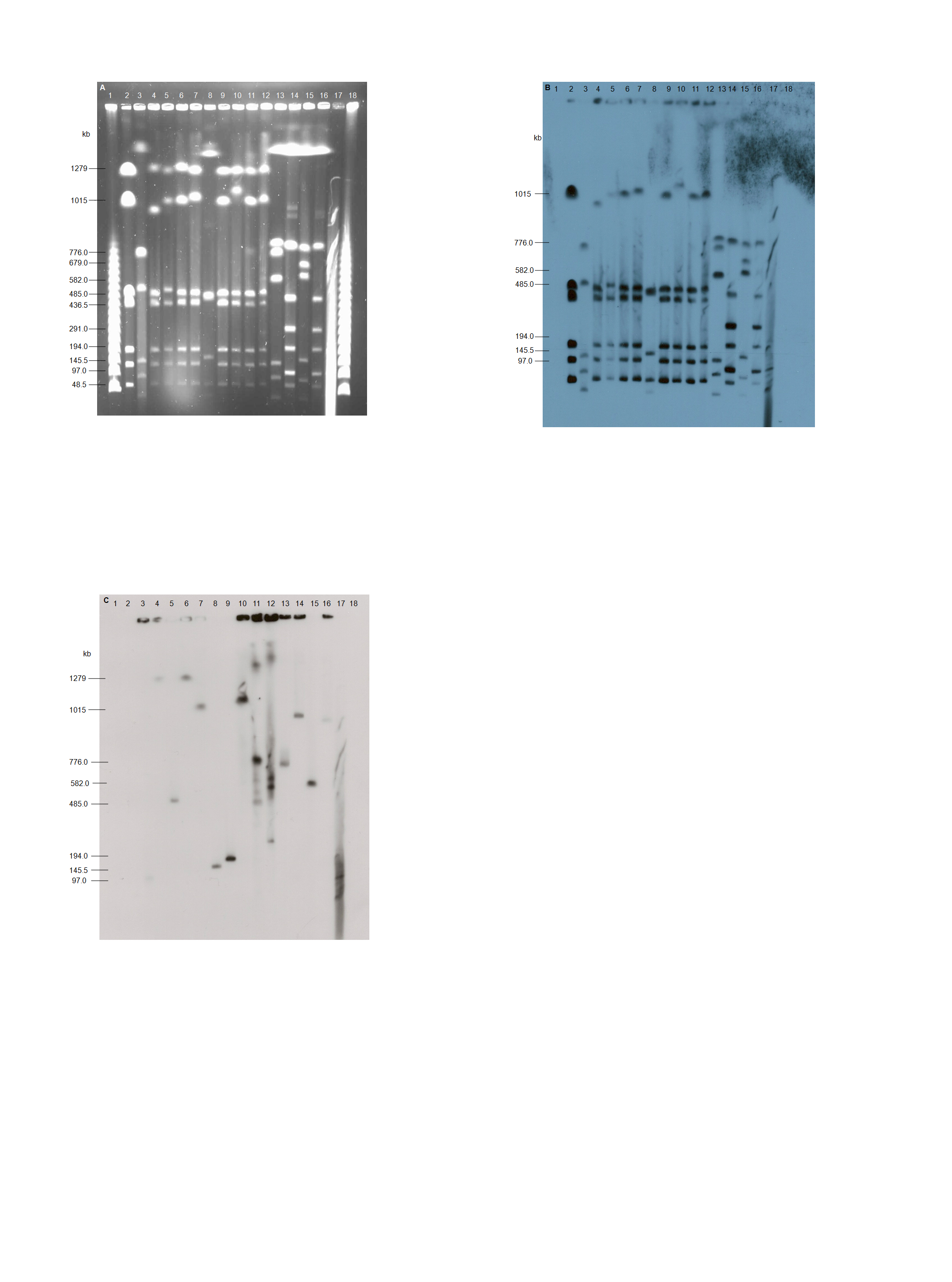

Supplement: Figure S3 — PFGE and Southern blot hybridization. A) Pulse-field gelelectrophoresis (PFGE) of I-CeuI-digested total DNA of recipient, donor and transformant bacteria; B) corresponding Southern hybridization with 16S rRNA probe; C) corresponding Southern hybridization with intI1 probe. Lane 1 – lambda PFG marker (New England Biolabs); 2 – A. baylyi BD413 (recipient); 3 – S. enterica serovar Typhimurium 490 (donor); 4–7 – transformants from exposure to DNA of S. enterica serovar Typhimurium 490, (St)1, SD1, (St)2, (St)3, respectively; 8 – A. baumannii 064 (donor); 9–12 – transformants from exposure to DNA of A. baumannii 064, SD2, (AbII)1, (AbII)2 and (AbII)3, respectively; 13 – S. enterica serovar Rissen 486 (donor); 14 – E. cloacae C2R371 (donor); 15 – E. coli C10R379 (donor); 16 – C. freundii C16R385 (donor); 17 – E. fergusonii AS041A2 (donor); 18 – lambda PFG marker (New England Biolabs). (TIF) [file ppat.1002837.s003.tif]

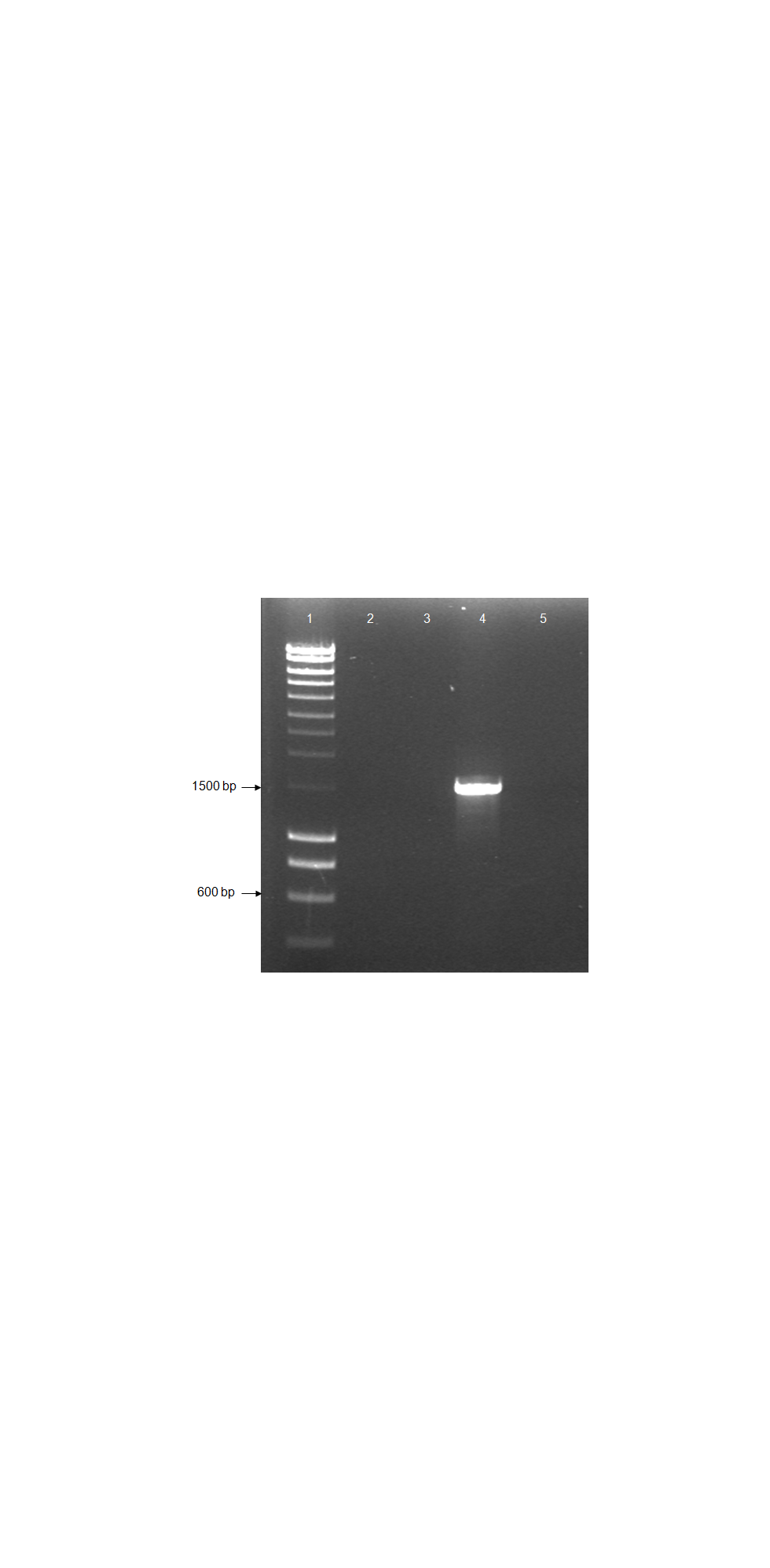

Supplement: Figure S4 — RT-PCR of the class 1 integrase RNA in transformant A. baylyi SD2. Lane 1 (left) – SmartLadder (Eurogentec); 2–3 –PCR targeting the intI1 gene, 2 – cDNA; 3 – RNA; 4–5 – PCR targeting the 16S rRNA gene, 4 – cDNA; 5 – RNA. For primers description, see Material and Methods. (TIF) [file ppat.1002837.s004.tif]
