# Supplementary material for: Natural Transformation Facilitates Transfer of Transposons, Integrons and Gene Cassettes between Bacterial Species
Source: PLoS Pathog. 2012 Aug 2;8(8):e1002837. doi: 10.1371/journal.ppat.1002837 (PMC3410848; doi:10.1371/journal.ppat.1002837)
Supplement: Table S1 — Antimicrobial susceptibility of A. baylyi transformants determined by the E-test method. (DOC) [file ppat.1002837.s005.doc]

**Table S1.** Antimicrobial susceptibility of *A. baylyi* transformants determined by the E-test method.

| Bacteria^a)^ | AM^b)^ | CAZ ^b)^ | CN ^b)^ | CTX ^b)^ | K ^b)^ | SC ^b)^ | SX ^b)^ | TOB ^b)^ |
| --- | --- | --- | --- | --- | --- | --- | --- | --- |
| *A. baylyi* BD413 | 1 | 1.5 | 0.064 | 1.5 | 0.38 | 3 | 16 | 0.5 |
| *A. baumannii* 064 | >256 | n.d. | >256 | n.d. | >256 | n.d. | >1024 | n.d. |
| *A. baylyi* SD2 | 0.75 | n.d. | 16 | n.d. | 96 | n.d. | >1024 | n.d. |
| (AbII)1 | 0.75 | n.d. | 48 | n.d. | 192 | n.d. | >1024 | n.d. |
| (AbII)2 | 1 | n.d. | 32 | n.d. | >256 | n.d. | >1024 | n.d. |
| (AbII)3 | 1 | n.d. | 64 | n.d. | >256 | n.d. | >1024 | n.d. |
| (SD2)1 | 0.75 | n.d. | 16 | n.d. | 64 | n.d. | >1024 | n.d. |
| (SD2)2 | 0.75 | n.d. | 24 | n.d. | 64 | n.d. | >1024 | n.d. |
| (SD2)3 | 0.75 | n.d. | 16 | n.d. | 64 | n.d. | >1024 | n.d. |
| (AbII)4 | 48 | n.d. | 0.38 | n.d. | 0.75 | n.d. | 16 | n.d. |
| (AbII)5 | 12 | n.d. | 0.5 | n.d. | 1 | n.d. | 32 | n.d. |
| (AbII)6 | 1 | n.d. | 0.38 | n.d. | <256 | n.d. | 48 | n.d. |
| (AbII)L1 | 32 | n.d. | 0.38 | n.d. | 0.5 | n.d. | 24 | n.d. |
| *A. baumannii* 65FFC | 64 | >256 | n.d. | >256 | n.d. | n.d. | >1024 | n.d. |
| *A. baylyi* SD3 | 8 | >256 | n.d. | >256 | 1.0 | n.d. | >1024 | n.d. |
| [SD2](AbI)1 | 8 | >256 | n.d. | >256 | 1.0 | n.d. | >1024 | n.d. |
| [SD2](AbI)2 | 8 | >256 | n.d. | >256 | 1.0 | n.d. | >1024 | n.d. |
| (SD3)1 | 6 | >256 | n.d. | >256 | n.d. | n.d. | >1024 | n.d. |
| (SD3)2 | 6 | >256 | n.d. | >256 | n.d. | n.d. | >1024 | n.d. |
| (SD3)3 | 6 | >256 | n.d. | >256 | n.d. | n.d. | >1024 | n.d. |
| (AbI)L1 | 6 | 8 | n.d. | 8 | n.d. | n.d. | 16 | n.d. |
| (AbI)L2 | 4 | 8 | n.d. | 8 | n.d. | n.d. | 64 | n.d. |
| (AbI)L3 | 0.75 | 1.5 | n.d. | 1.5 | n.d | n.d. | 24 | n.d. |
| [KOI](AbI)1 | 6 | >256 | n.d. | >256 | 1.5 | n.d. | >1024 | n.d. |
| [KOI](AbI)2 | 6 | >256 | n.d. | >256 | 1.5 | n.d. | >1024 | n.d. |
| [KOI](AbI)3 | 6 | >256 | n.d. | >256 | 1.5 | n.d. | >1024 | n.d. |
| [RAM](AbI)1 | 4 | >256 | n.d. | 64 | 1.0 | n.d. | >1024 | n.d. |
| [RAM](AbI)2 | 4 | >256 | n.d. | 64 | 1.0 | n.d. | >1024 | n.d. |
| [RAM](AbI)3 | 4 | >256 | n.d. | 64 | 1.0 | n.d. | >1024 | n.d. |
| *C. freundii* C16R385 | n.d. | n.d. | n.d. | n.d. | n.d. | 192 | >1024 | n.d. |
| *A. baylyi* [SD2](Cf)1 | n.d. | n.d. | n.d. | n.d. | 1.5 | 64 | >1024 | n.d. |
| [SD2](Cf)2 | n.d. | n.d. | n.d. | n.d. | 1.5 | 96 | >1024 | n.d. |
| [SD2](Cf)3 | n.d. | n.d. | n.d. | n.d. | 1.5 | 16 | >1024 | n.d. |
| *E. cloacae* C2R371 | n.d. | n.d. | n.d. | n.d. | n.d. | 192 | >1024 | n.d. |
| *A. baylyi* [SD2](Ecl)1 | n.d. | n.d. | n.d. | n.d. | 1.5 | >256 | >1024 | n.d. |
| [SD2](Ecl)2 | n.d. | n.d. | n.d. | n.d. | 1.0 | >256 | >1024 | n.d. |
| [SD2](Ecl)3 | n.d. | n.d. | n.d. | n.d. | 1.0 | 96 | >1024 | n.d. |
| *E. coli* C10R379 | n.d. | n.d. | n.d. | n.d. | n.d. | n.d. | >1024 | n.d. |
| *A. baylyi* [SD2](EcI)1 | n.d. | n.d. | n.d. | n.d. | 1.5 | n.d. | >1024 | n.d. |
| [SD2](EcI)2 | n.d. | n.d. | n.d. | n.d. | 1.5 | n.d. | >1024 | n.d. |
| [SD2](EcI)3 | n.d. | n.d. | n.d. | n.d. | 1.5 | n.d. | >1024 | n.d. |
| *E. coli* K71-77 | >256 | >256 | >1024 | >256 | n.d. | n.d. | >1024 | >256 |
| *A. baylyi* (EcII)1 | 3 | n.d. | >256 | 6 | n.d. | n.d. | 256 | 16 |
| (EcII)2 | 3 | n.d. | 64 | 6 | n.d | n.d. | 256 | 64 |
| (EcII)3 | 3 | n.d. | >256 | 6 | n.d. | n.d. | 256 | 128 |
| (EcII)4 | 3 | n.d. | >256 | 6 | n.d. | n.d. | 256 | 12 |
| *E. fergusonii* AS041A2 | n.d. | n.d. | n.d. | n.d. | n.d. | 8 | >1024 | n.d. |
| *A. baylyi* [SD2](Ef)1 | n.d. | n.d. | n.d. | n.d. | 1.5 | >1024 | >1024 | n.d. |
| [SD2](Ef)2 | n.d. | n.d. | n.d. | n.d. | 1.5 | >1024 | >1024 | n.d. |
| [SD2](Ef)3 | n.d. | n.d. | n.d. | n.d. | 1.5 | 96 | >1024 | n.d. |
| *K. pneumoniae* K66-45 | >256 | >256 | >1024 | >256 | n.d. | n.d. | >1024 | >256 |
| *A. baylyi* (Kp)1 | 3 | n.d. | >256 | 6 | n.d. | n.d. | >1024 | >256 |
| (Kp)2 | 3 | n.d. | >256 | 6 | n.d. | n.d. | >1024 | >256 |
| *P. aeruginosa* SM | >256 | n.d. | 16 | n.d. | n.d. | >1024 | >1024 | n.d. |
| *A. baylyi* SD6 | >256 | n.d. | 1 | n.d. | 12 | 256 | >1024 | n.d. |
| [SD2](Ps)1 | 16 | n.d. | 0.5 | n.d. | 2 | 24 | >1024 | n.d. |
| [SD2](Ps)2 | >256 | n.d. | 1.5 | n.d. | 24 | 512 | >1024 | n.d. |
| (SD6) 1 | >256 | n.d. | 1 | n.d. | n.d. | 192 | >1024 | n.d. |
| (SD6) 2 | >256 | n.d. | 1 | n.d. | n.d. | 512 | >1024 | n.d. |
| (SD6) 3 | >256 | n.d. | 1 | n.d. | n.d. | 384 | >1024 | n.d. |
| (Ps)1 | 6 | n.d. | 0.19 | n.d. | n.d. | 3 | 24 | n.d. |
| (Ps)L1 | 6 | n.d. | 0.5 | n.d. | n.d. | 4 | 24 | n.d. |
| (Ps)L2 | 6 | n.d. | 0.38 | n.d. | n.d. | 3 | 24 | n.d. |
| [KOI](Ps)1 | 48 | n.d. | 0.75 | n.d. | 3 | 128 | >1024 | n.d. |
| [KOI](Ps)2 | >256 | n.d. | 0.75 | n.d. | 4 | 192 | >1024 | n.d. |
| [KOI](Ps)3 | >256 | n.d. | 3 | n.d. | 48 | >1024 | >1024 | n.d. |
| [RAM](Ps)1 | >256 | n.d. | 0.75 | n.d. | 6 | 384 | >1024 | n.d. |
| [RAM](Ps)2 | 48 | n.d. | 0.25 | n.d. | 2 | 32 | >1024 | n.d. |
| [RAM](Ps)3 | 32 | n.d. | 0.25 | n.d. | 2 | 32 | >1024 | n.d. |
| *S. enterica* Rissen 486 | >256 | n.d. | n.d. | n.d. | n.d. | >1024 | >1024 | n.d. |
| *A. baylyi* SD5 | 1.5 | n.d. | n.d. | n.d. | 1.0 | 24 | >1024 | n.d. |
| [SD2](Sr)1 | 1 | n.d. | n.d. | n.d. | 1.0 | 96 | >1024 | n.d. |
| [SD2](Sr)2 | 1 | n.d. | n.d. | n.d. | 1.0 | 64 | >1024 | n.d. |
| (SD5)1 | 1 | n.d. | n.d. | n.d. | n.d. | 48 | >1024 | n.d. |
| (SD5)2 | 1 | n.d. | n.d. | n.d. | n.d. | 32 | >1024 | n.d. |
| (SD5)3 | 0.75 | n.d. | n.d. | n.d. | n.d. | 32 | >1024 | n.d. |
| (Sr)1 | 4 | n.d. | n.d. | n.d. | n.d. | 3 | 16 | n.d. |
| [KOI](Sr)1 | 3 | n.d. | n.d. | n.d. | 1.0 | >1024 | >1024 | n.d. |
| [KOI](Sr)2 | 3 | n.d. | n.d. | n.d. | 1.0 | >1024 | >1024 | n.d. |
| [KOI](Sr)3 | 2 | n.d. | n.d. | n.d. | 1.0 | >1024 | >1024 | n.d. |
| *S. enterica* Typh. 490 | >256 | n.d. | n.d. | n.d. | n.d. | 192 | >1024 | n.d. |
| *A. baylyi* (St)1 | 32 | n.d. | n.d. | n.d. | n.d. | 12 | >1024 | n.d. |
| SD1 | 6 | n.d. | n.d. | n.d. | n.d. | 12 | >1024 | n.d. |
| (St)2 | 3 | n.d. | n.d. | n.d. | n.d. | 8 | >1024 | n.d. |
| (St)3 | 12 | n.d. | n.d. | n.d. | n.d. | 32 | >1024 | n.d. |
| SD4 | 8 | n.d. | n.d. | n.d. | 1.0 | 16 | >1024 | n.d. |
| [SD2](St)1 | 16 | n.d. | n.d. | n.d. | 1.0 | 96 | >1024 | n.d. |
| [SD2](St)2 | 4 | n.d. | n.d. | n.d. | 1.0 | 16 | >1024 | n.d. |
| (SD1)1 | 6 | n.d. | n.d. | n.d. | n.d. | 12 | >1024 | n.d. |
| (SD1)2 | 4 | n.d. | n.d. | n.d. | n.d. | 12 | >1024 | n.d. |
| (SD1)3 | 6 | n.d. | n.d. | n.d. | n.d. | 16 | >1024 | n.d. |
| (SD4)1 | 6 | n.d. | n.d. | n.d. | n.d. | 16 | >1024 | n.d. |
| (SD4)2 | 4 | n.d. | n.d. | n.d. | n.d. | 12 | >1024 | n.d. |
| (SD4)3 | 6 | n.d. | n.d. | n.d. | n.d. | 16 | >1024 | n.d. |
| (St)L1 | 6 | n.d. | n.d. | n.d. | n.d. | 3 | 64 | n.d. |
| (St)L2 | 6 | n.d. | n.d. | n.d. | n.d. | 3 | 32 | n.d. |
| [KOI](St)1 | 16 | n.d. | n.d. | n.d. | 1.0 | 48 | >1024 | n.d. |
| [KOI](St)2 | >256 | n.d. | n.d. | n.d. | 1.0 | >1024 | >1024 | n.d. |
| [KOI](St)3 | >256 | n.d. | n.d. | n.d. | 1.0 | >1024 | >1024 | n.d. |
| [RAM](St)1 | 12 | n.d. | n.d. | n.d. | 0.5 | 24 | >1024 | n.d. |
| [RAM](St)2 | 12 | n.d. | n.d. | n.d. | 0.5 | 16 | >1024 | n.d. |
| [RAM](St)3 | 12 | n.d. | n.d. | n.d. | 0.5 | 24 | >1024 | n.d. |
| ^a^ For each *A. baylyi* transformant, the name is given by the code [recipient](donor)isolate number. Whenever the recipient is not mentioned, the *A. baylyi* BD413 strain was used; AbI – *Acinetobacter baumannii* 65FFC; AbII – *Acinetobacter baumannii* 064; Cf – *Citrobacter freundii* C16R385; Ecl – *Enterobacter cloacae* C2R371; EcI – *Escherichia coli* C10R379; EcII - *Escherichia coli* K71-77; Ef – *Escherichia fergusonii* AS041A2; Kp – *Klebsiella pneumoniae* K66-45; Ps – *Pseudomonas aeruginosa* SM; Sr – *Salmonella enterica* serovar Rissen 486; St – *Salmonella enterica* serovar Typhimurium 490.  ^b^ Antibiotics AM – ampicillin; CAZ – ceftazidime; CN – gentamicin; CTX – cefotaxime; K – kanamycin; SC – spectinomycin; SX – sulphamethoxazole; and TOB – tobramycin. | | | | | | | | |
